# Novel small molecule 11*β*-HSD1 inhibitor from the endophytic fungus *Penicillium commune*

**DOI:** 10.1038/srep26418

**Published:** 2016-05-19

**Authors:** Weiguang Sun, Xintao Chen, Qingyi Tong, Hucheng Zhu, Yan He, Liang Lei, Yongbo Xue, Guangmin Yao, Zengwei Luo, Jianping Wang, Hua Li, Yonghui Zhang

**Affiliations:** 1 Hubei Key Laboratory of Natural Medicinal Chemistry and Resource Evaluation, School of Pharmacy, Tongji Medical College, Huazhong University of Science and Technology, Wuhan 430030, China; 2 Puai Hospital Affliated to Tongji Medical College, Huazhong University of Science and Technology, Wuhan 430030, People’s Republic of China

## Abstract

Two new phenone derivatives penicophenones A (**1**) and B (**2**), a new cyclic tetrapeptide penicopeptide A (**3**), and five known compounds were isolated from the culture broth of *Penicillium commune*, an endophytic fungus derived from *Vitis vinifera*. Compounds **1**–**3** were elucidated by extensive spectroscopic analyses including 1D and 2D NMR and HRESIMS. The absolute configurations of **1** and **3** were determined by comparing its ECD with related molecules and modified Marfey’s analysis, respectively. Penicophenone A (**1**) possesses a rare benzannulated 6,6-spiroketal moiety, which is a new member of the unusual structural class with peniphenone A as the representative. Compound **3** exhibited significant inhibition activities against 11*β*-hydroxysteroid dehydrogenase type 1 (11*β*-HSD1) *in vitro* and showed strong binding affinity to 11*β*-HSD1. Moreover, compound **3** treatments decreased the lipid droplet accumulation associate with the inhibition of 11*β*-HSD1 expression in differentiate-induced 3T3-L1 preadipocytes. Furthermore, the molecular docking demonstrated that compound **3** coordinated in the active site of 11*β*-HSD1 is essential for the ability of diminishing the enzyme activity.

Glucocorticoids influence a wide variety of physiologic processes, including lipid and glucose metabolism, immune modulation, cell growth, and anti-inflammatory responses. Excessive activation of glucocorticoid hormones (GCs) can result in metabolic syndromes with multiple clinical features, including insulin resistant diabetes, obesity, dyslipidemia, and hypertension^1^. 11*β*-hydroxysteroid dehydrogenase type 1 (11*β*-HSD1), which converts inactive glucocorticoid into active glucocorticoid, such as cortisol in humans can amplify the local levels and activities of GCs^2^. Given that 11*β*-HSD1 is abundantly expressed in metabolically important tissues which become resistant to insulin action in type 2 diabetes, such as adipose, muscle, and liver tissue, the inhibition of 11*β*-HSD1 offers the ability to restore the metabolic action of insulin in these tissues^3^. Consequently, the developments of 11*β*-HSD1 inhibitor drugs are urgently demanded.

Natural products (NPs) have represented a cornerstone of pharmaceutical research, as they offer a diverse range of bioactive substructures, chemical scaffolds, and potentially lower toxicity profiles^4^. As the literature reported, more than 50% of new medicines approved by the US Food and Drug Administration (FDA) between 1981 and 2014 were direct or indirect derived from NPs^5^. Encouraged by this, our research group has been dedicated to explore new NPs targeting 11*β*-HSD1 in recent years. Fungi are rich sources of novel secondary metabolites with diverse structures and various biological activities, which have attracted considerable attentions. In our search for new bioactive substances from fungi^6^,^7^,^8^,^9^,^10^,^11^, the fungus *Penicillium commune* was phytochemically investigated, which led to the isolation of two new phenone derivatives penicophenones A (**1**) and B (**2**), a new cyclic tetrapeptide penicopeptide A (**3**), and five known compounds 3-benzylidene-3,4-dihydro-4-methyl-lH-l,4-benzodiazepine-2,5-dione (**4**)^12^, (+)-cyclopenol (**5**)^13^, cyclopenin (**6**)^13^,^14^, emindole SB (**7**)^15^, and penilactones A (**8**)^16^ (Fig. 1). Compounds **1**–**8** were subjected to 11*β*-HSD1 inhibitory assays, and compound **3** exhibited significant inhibition activities against 11*β*-HSD1 *in vitro* and showed strong binding affinity to recombinant human 11*β*-HSD1 by microscale thermophoresis (MST) assays. Moreover, compound **3** treatments decreased the lipid droplet accumulation associate with the inhibition of 11*β*-HSD1 activity of 3T3-L1 cells *in vivo*. Furthermore, the molecular docking demonstrated that compound **3** is able to occupy the major active site and adopt a conformation similar to the known inhibitor, carbenoxolone.

## Results and Discussion

### Isolation and Structure Elucidation

The strain of *P.commune* was cultured on rice at 28 °C for 31 days, and then extracted with EtOAc to yield a crude extract. The extract was suspended in H_2_O and partitioned successively with petroleum ether and EtOAc to yield a petroleum ether-soluble extract and a EtOAc-soluble extract. The petroleum ether-soluble part was separated and purified to obtain compounds **1** (8.1 mg), **6** (7.5 mg), and **8** (7.9 mg). The EtOAc extract was also separated and purified to yield compounds **2** (9.6 mg), **3** (40.7 mg), **4** (36.0 mg), **5** (40 mg), and **7** (23 mg)

Penicophenone A (**1**) was isolated as colorless oil. The molecular formula of C_18_H_24_O_5_ was deduced by HRESIMS (*m/z* 321.1694 [M + H]^+^, calcd for C_18_H_25_O_5_, 321.1702) and ^13^C NMR data, suggesting seven degrees of unsaturation. The ^1^H NMR (Table 1) spectrum of **1** showed an aromatic proton (*δ*_H_ 7.28, s), two oxygenated methine protons (*δ*_H_ 4.00, m; 3.61, dqd, *J* = 12.5, 6.2, 2.1 Hz), six aliphatic protons (*δ*_H_ 2.69, dd, *J* = 16.8, 6.4 Hz; 2.20, dd, *J* = 16.8, 2.8 Hz; 2.13, ddd, *J* = 12.6, 4.8, 1.7 Hz; 1.81, m; 1.09, dd, *J* = 12.6, 11.3 Hz; and 0.99, dt, *J* = 12.1, 11.6 Hz), and four methyls (*δ*_H_ 2.33, s; 1.94, s; 0.87, d, *J* = 6.3 Hz; and 0.73, d, *J* = 7.1 Hz). The ^13^C NMR data (Table 1) revealed that **1** has a skeleton based on 18 carbons, including a carbonyl (*δ*_C_ 204.8), six aromatic carbons (*δ*_C_ 110.1–161.9), a hemiketal carbon (*δ*_C_ 102.9), two oxygenated methines (*δ*_C_ 67.7 and 65.3), four aliphatic carbons (*δ*_C_ 34.5–24.5), and four methyls (*δ*_C_ 15.4–26.3).

The planar structure was established by analyses of its ^1^H–^1^H COSY and HMBC spectra (Fig. 2). The pentasubstituted benzene ring (ring A) was constructed by HMBC correlations from H-7 to C-1, C-5, and C-6, from Me-14 to C-1, C-2, and C-3, and from H-3 to C-1 and C-5 based on the former mentioned six aromatic carbons. Additional HMBC cross-peaks from Me-16 to C-4 and C-15 and from H-3 to C-15 supported the location of the acetyl at C-4. The ^1^H–^1^H COSY spectrum showed the presence of two spin systems of H-7/H-8/Me-17 and H-10/H-11/H-12/H-13/Me-18, which were connected to the hemiketal carbon by HMBC interactions from H-7, H-10, Me-17, and Me-18 to C-9 and from H-10 to C-8. The chemical shifts of C-1 (*δ*_C_ 157.3) and C-5 (*δ*_C_ 161.9), together with degrees of unsaturation requirement, indicated the presence of an additional pyran ring (ring B) between C-1 and C-9 via an oxygen atom. Therefore, the planar structure of **1**, with a rare benzannulated 6,6-spiroketal moiety was determined.

The relative configuration of **1** was determined by a NOESY experiment (Fig. 2). NOESY cross-peak observed between H-10*β* and H-13 established a boat conformation of ring C. The *α*-orientation of the hydroxyl at C-11 was revealed by NOESY cross-peak of H-11/H-13. In addition, NOESY correlations of H-10*β*/Me-17 and H-10*α*/H-8 not only determined the configuration of the spiroketal carbon (C-9) but also clarified the *α*-orientation of Me-17. The absolute configuration of **1** was finally determined as 8*S*,9*R*,11*S*,13*R* by comparing its ECD spectrum (see Supplementary Information) with that of peniphenone A^17^, because both of them exhibit negative cotton effects around 230 and 280 nm. Thus, compound **1** determined to be a novel compound possessing a rare benzannulated 6,6-spiroketal core structure, which is a new member of the unusual structural class with peniphenone A as the representative. The biosynthetic pathway of **1** may be similar to that of peniphenone A with a different precursor of pyrone derivative (5-hydroxy-6-methyl-pyrone in **1** rather than 5-hydroxy-3,6-dimethyl-pyrone in peniphenone A), which lead to compound **1** with the absence of methyl group at C-10 compared with that of peniphenone A.

**Figure 1:**
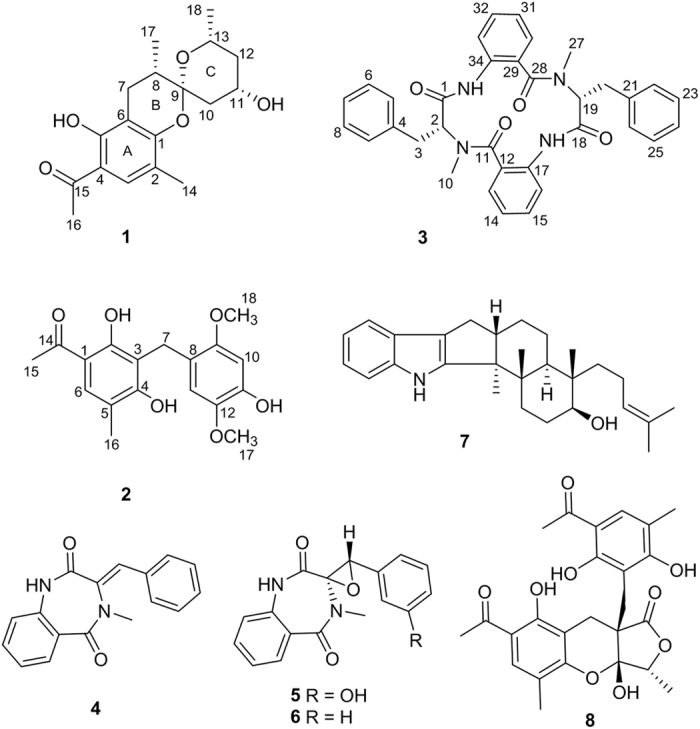
Structures of 1–8.

**Figure 2:**
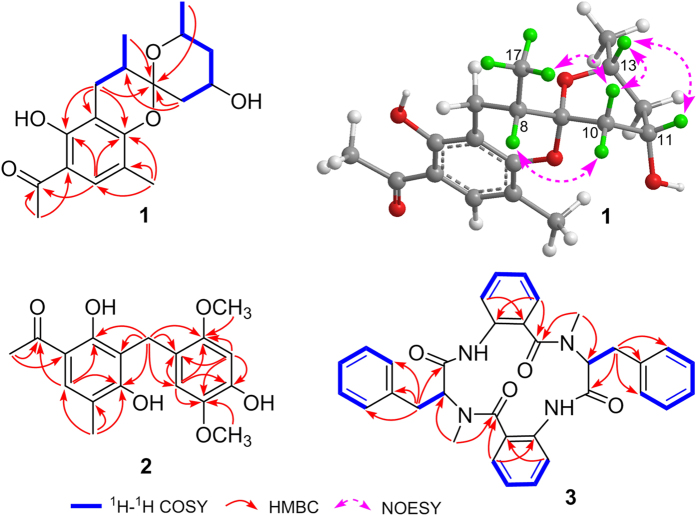
^1^H–^1^H COSY and selected HMBC of **1**–**3** and key NOESY of **1**.

Penicophenone B (**2**) was obtained as yellowish oil, with the molecular formula of C_18_H_20_O_6_ suggesting by HRESIMS data ([M]^+^ *m/z* 332.1254, calcd for C_18_H_20_O_6_, 332.1260). The ^1^H and ^13^C NMR spectra of **2** showed resonances of a carbonyl, 12 aromatic carbons (including three methines), a methylene, two methyls, and two methoxyls. Extensive analysis of the HMBC (Fig. 2) spectrum of **2** revealed that it had the same 2,4-diol-5-methyl acetophenone as that of **1**. The remained tetrasubstituted benzene ring, including the location of the methoxyls, were elucidated by HMBC correlations from H-10 and H-13 to C-8, C-9, C-11, and C-12, from OMe-17 to C-12, and from OMe-18 to C-9. The linkage of the former established two rings via the methylene were further confirmed by HMBC correlations from H-7 to C-2, C-3, C-4, C-8, C-9, and C-13. Herein, the structure of **2** was elucidated as shown in Fig. 1.

Penicopeptide A (**3**) was assigned the molecular formula of C_34_H_32_N_4_O_4_ on the basis of its HRESIMS data ([M + Na]^+^ *m/z* 583.2310, calcd for C_34_H_32_N_4_O_4_Na, 583.2321). The peptide nature of **3** was inferred from the presence of signals of the amide N-Me (*δ*_H_ 2.90, s; 3.07, s and *δ*_C_ 39.8; 29.6) and amino acid protons (*δ*_H_ 4.33, dd, *J* = 10.6, 7.0 Hz and 4.43, t, *J* = 7.6 Hz) (Table 2). Careful interpretation of the 2D NMR data revealed the presence of two phenylalanine (Phe) and two 2-aminobenzoic acid residues. The connections of the above mentioned residues were established by HMBC (Fig. 2) interactions from Me-10 to C-2 and C-11, H-2 to C-11, Me-27 to C-19 and C-28, and H-19 to C-28 aided by the HRESIMS data. The absolute configurations of C-2 and C-19 in the Phe units were determined to be *R* by modified Marfey’s analysis (see Supplementary Information). Compound **3** is a symmetrical tetrapeptide, and normally its ^1^H and ^13^C NMR data of two units should typically overlaped; however this is not observed in this case. The main reason may be that the conformations of **3** are asymmetrical, which is supported by the conformational analysis (see Supplementary Information).

The structure of 3-benzylidene-3,4-dihydro-4-methyl-lH-l,4-benzodiazepine-2,5-dione (**4**) was firstly determined by single crystal X-ray analysis (CCDC 1058389) in this study. In addition, its ^1^H and ^13^C NMR data were also provided.

### Inhibition of compounds 1-8 on 11*β*-HSD1 enzyme activity

**Figure 3:**
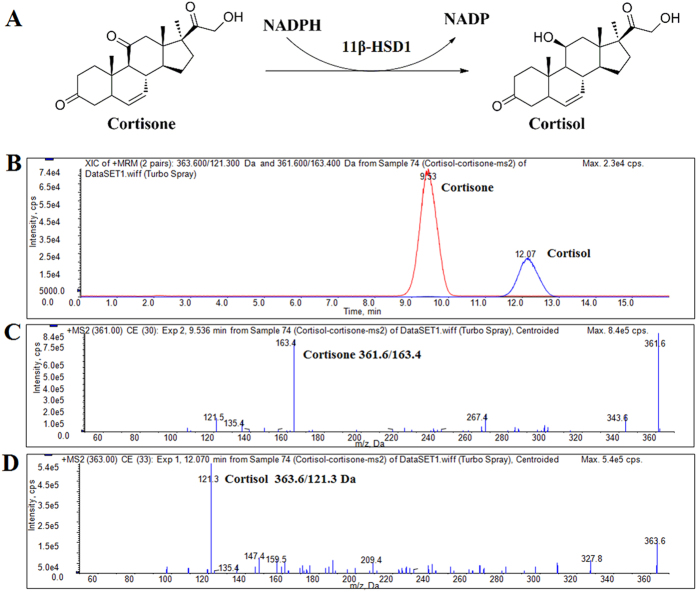
Determination of cortisone and cortisol by HPLC-MS/MS. (**A**) Biological activity of 11*β*-HSD1. (**B**) HPLC-MS/MS Chromatogram of cortisone and cortisol, (**C,D**) Positive ESI MS/MS spectra acquired on an API4000 mass spectrometer for cortisol with collision energy of 33 eV and cortisone with collision energy of 30 eV.

To explore the 11*β*-HSD1 inhibitory activities of compounds **1**–**8** *in vitro*, recombinant human 11*β*-HSD1 protein was incubated with various concentrations of compounds and the enzymatic activity (cortisone to cortisol) was tested using HPLC-MS/MS (Fig. 3). Among these compounds tested, compound **3** showed strong inhibitory effect against human 11*β*-HSD1, with an IC_50_ value of 9.07 ± 0.61 *μ*M (Table 3).

### Specific binding with 11*β*-HSD1

**Figure 4:**
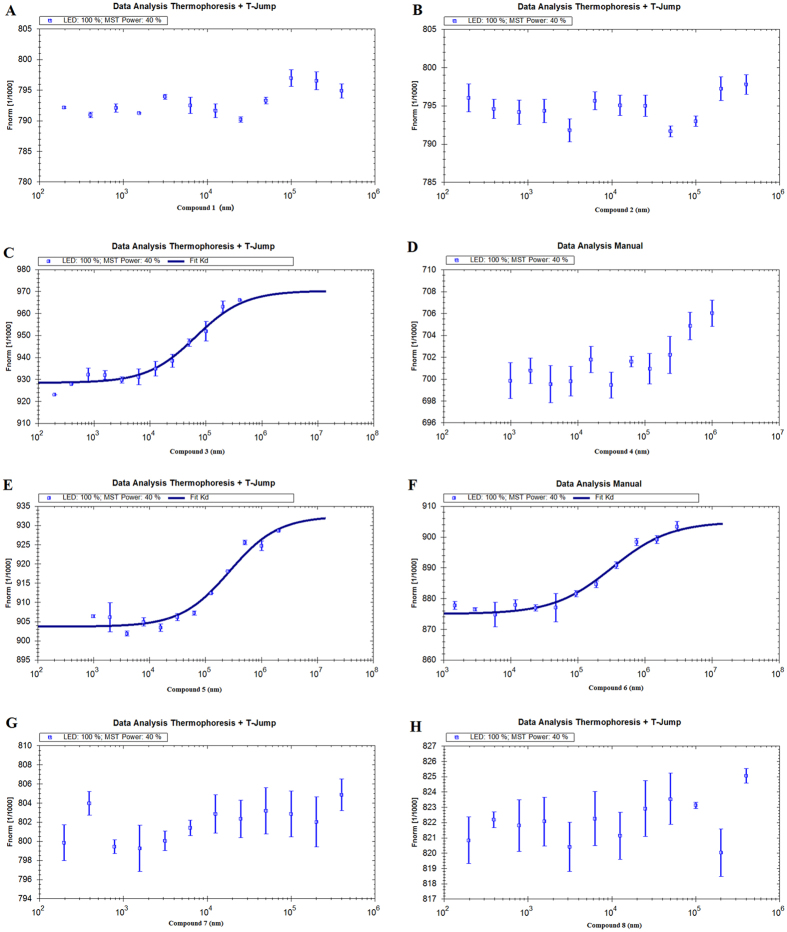
Measurement of affinity between compounds 1–8 with 11*β*-HSD1 by MST. The resulting binding curve was shown. From the resulting binding curve, Kd of 82.1 ± 5.01 *μ*M for **3** (**C**), 420.51 ± 12.11 *μ*M for **5** (**E**), 513.14 ± 10.61 *μ*M for **6** (**F**) were calculated.

Measuring the thermophoretic behaviour of a protein in the presence of differing ligand concentrations by MST allows quantitative analysis of molecular interactions in solution. The MST technique has previously been used to investigate protein-protein, small organic molecule-protein and antibody-protein interactions. In this study, MST was utilized as an independent confirmation of the dissociation constant (Kd) between **1**–**8** and 11*β*-HSD1. As shown in Table 3, it is not surprising that the Kd value of compound **3** (82.1 ± 5.01 *μ*M) was much lower than that of 5 and 6, and similarly, either no binding or no statistically significant binding was detected when 11*β*-HSD1 was titrated with compounds **1**, **2**, **4**, **7**, and **8** (Fig. 4). These results confirmed the specific binding of compound **3** to 11*β*-HSD1.

### Effect of compound 3 on lipid accumulation and 11*β*-HSD11 expression in 3T3-L1 cells

**Figure 5:**
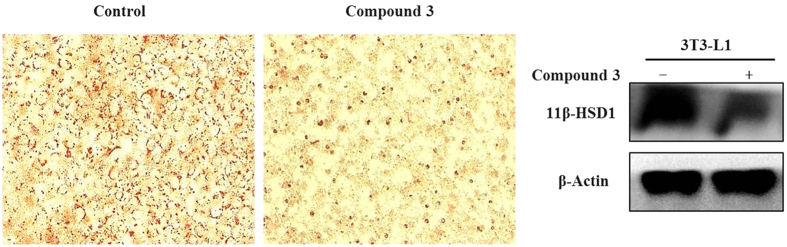
Effects of compound **3** on lipid accumulation and 11*β*-HSD1 expression in 3T3-L1 cells. Preadipocytes were induced to differentiate in the presence or absence of compound **3**, which was then stained with Oil Red O and photographed (40×). 11*β*-HSD1 expressions were determined by western blot. *β*-Actin was used as a loading control.

It was reported that 11*β*-HSD1 is highly expressed in normal liver^18^ and stimulates pre-adipocyte differentiation. As a result, many 11*β*-HSD1 inhibitors were investigated to prevent adipogenesis^19^. To further examine the 11*β*-HSD1 inhibitory effects of compound **3**, we examined the anti-adipogenic effect of compound **3** during the differentiation of 3T3-L1 cells. As the results shown in Fig. 5, compound **3** treatments decreased the lipid droplet accumulation associate with the inhibition of 11*β*-HSD1 activities.

### Molecular docking analysis

**Figure 6:**
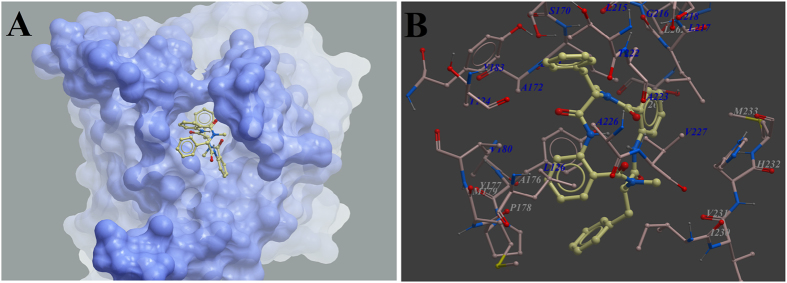
Low-energy binding conformations of compound 3 bound to 11*β*-HSD1 generated by virtual ligand docking. Compound **3** as the ball-and-stick model showing carbon (yellow), hydrogen (grey), oxygen (red) and Nitrogen (blue) atoms. (**A**) Compound **3** was observed to occupy the active site with significant scores of ICM docking score and IMC docking mfScore, and adopted a conformation similar to that of known inhibitors. (**B**) Compound **3** had the ability to form key hydrophobic interaction with residues Leu126, Tyr177, Tyr183, and Leu171.

11*β*-HSD1 as the target of compound **3** was further evaluated by 3D molecular docking, utilizing the X-ray crystal structure of 11*β*-HSD1 (PDB code: 2BEL). Using the Molsoft ICM method (ICM-Pro 3.8 molecular docking software), we optimized the geometry of compound **3** and determined the binding site with the lowest-energy and the most favorable orientation of the compound. Compound 3 was observed to occupy the major active site with significant scores of ICM docking score and IMC docking mfScore, and adopted a conformation similar to that of known inhibitor (Fig. 6A). Compound **3** had the ability to form key hydrophobic interactions with residues Leu126, Tyr177, Tyr183, and Leu171 (Fig. 6B).

To date, several small molecule inhibitors of 11*β*-HSD1 were discovered from NPs, such as glycyrrhetinic acid, carbenoxolone, gossypol, and estradiol. However, these NPs often showed low specificity in the inhibition of 11*β*-HSD1 with serious side effects. Thus, more efforts to find new 11*β*-HSD1-targeting candidates are urgently demanded^20^,^21^. In this study, a new cyclic tetrapeptide penicopeptide A (**3**) was isolated from the fungus of *Penicillium commune*, which showed the most potent and concentration-dependent inhibitions activity with an IC_50_ value of 9.07 ± 0.61 *μ*M. The possible mechanism may be that compound **3** is more accessible to the active site of 11*β*-HSD1 to exert selective inhibition effects, which is indicated by MST assay (Kd = 82.1 ± 5.01 *μ*M). Furthermore, the molecular docking demonstrates that compound **3** coordinating in the active site of 11*β*-HSD1 is essential for the ability of diminishing the enzyme activity. In summary, the novel structure of compound **3** combined with its significant inhibitory activities of 11*β*-HSD1 reported in this study may greatly promote the studies of 11*β*-HSD1 inhibitors from natural products, and further investigations on the mechanism and structure-function relationships for developing more excellent agents are necessary.

## Methods

### General experimental procedures

Optical rotations were determined with a Perkin-Elmer 41 polarimeter equipped with a sodium lamp (589 nm). UV, CD, and FT-IR spectra were performed on a Varian Cary 50, a JASCO-810 CD spectrometer, and a Bruker Vertex 70 instruments, respectively. 1D and 2D NMR spectra were recorded on a Bruker AM-400 spectrometer, and the ^1^H and ^13^C NMR chemical shifts were referenced with respect to the solvent or solvent impurity peaks. HRESIMS were carried out in the positive ion mode on a Thermo Fisher LC-LTQ-Orbitrap XL spectrometer. X-ray data were collected using a Bruker APEX DUO instrument. Column chromatography was conducted with silica gel (200–300 and 300–400 mesh; Qingdao Marine Chemical Inc., China), Sephadex LH-20 (GE Healthcare Bio-Sciences AB, Sweden), and MCI gel (75–150 *μ*m, Mitsubishi Chemical Corporation, Tokyo, Japan). Semi-preparative HPLC was carried out on a Dionex quaternary system with a diode array detector at a flow rate of 2.5 mL/min using a reversed-phased C_18_ column (5 *μ*m, 10 × 250 mm, YMC-pack ODS-A).Thin-layer chromatography (TLC) was performed with silica gel 60 F_254_ (Yantai Chemical Industry Research Institute) and RP-C_18_ F_254_ plates (Merck, Germany).

### Fungal material

The fungal strain *P.commune* was isolated as an endophytic fungus from the plant *Vitis vinifera*, which was collected at Qingshui County in Gansu province of China, in July 2007. A voucher specimen (No. 20140301) has been deposited in the culture collection center of Tongji Medical College, Huazhong University of Science and Technology.

### Fermentation, extraction, and isolation

The strain of *P.commune* was cultured with rice at 28 °C for 31 days, and then extracted with EtOAc to yield a crude extract (172 g). The extract was suspended in H_2_O and partitioned successively with petroleum ether and EtOAc to yield a petroleum ether-soluble extract (94 g) and a EtOAc-soluble extract (48 g). The petroleum ether-soluble part (94 g) was separated into five fractions (Fr.1−Fr.5) via silica gel column chromatography (CC, 1.5 kg, 10 × 100 cm) eluted with gradient petroleum ether/acetone (50:1 → 1:1). Compounds **1** (8.1 mg), **6** (7.5 mg), and **8** (7.9 mg) were purified from Fr.4 by repeated silica gel and ODS CC (MeOH/H_2_O, 30:70 → 90:10). The EtOAc extract (48 g) was subjected to silica gel CC (800 g, 10 × 100 cm) with gradient petroleum ether/acetone (20:1 → 1:2) to give seven fractions (Fr.A–Fr.G). Fr.C was further purified by repeated silica gel CC and semi-preparative HPLC (MeOH/H_2_O, 70:30) to give compounds **2** (9.6 mg) and **7** (23 mg). Purification of Fr.D by MPLC (MeOH/H_2_O, 30:70 → 70:30) followed by semi-preparative HPLC (MeOH/H_2_O, 65:35) led to the isolation of **3** (40.7 mg) and **4** (36.0 mg). Fr.F was also purified by MPLC (MeOH/H_2_O, 30:70 → 70:30) followed by semi-preparative HPLC to give **5** (40 mg).

*Penicophenone A (**1**)* Colorless oil; [α]^20^_D_ + 6.0 (c = 0.44, MeOH); UV (MeOH) *λ*_max_ (log ε) = 216 (4.01), 282 (3.87), 328 (3.47) nm; IR *ν*_max_ = 3392, 1627, 1478, 1447, 1382, 1179, 1136, 1072 cm^–1^; CD (MeOH) *λ*_max_ (Δε) 229 (−3.0), 280 (−1.5) nm; For ^1^H NMR (400 MHz) and ^13^C NMR (100 MHz) data see Table 1; HRESIMS [M + H]^ + ^*m/z* 321.1694 (calcd for C_18_H_25_O_5_, 321.1702).

*Penicophenone B (**2**)* Yellowish oil; UV (MeOH) *λ*_max_ (log ε) = 205 (4.72), 288 (4.05) nm; IR *ν*_max_ = 3216, 1689, 1623, 1483, 1453, 1395, 1199 cm^–1^; For ^1^H NMR (400 MHz) and ^13^C NMR (100 MHz) data see Table 1; HRESIMS [M]^+^ *m/z* 332.1254 (calcd for C_18_H_20_O_6_, 332.1260).

*Penicopeptide A (**3**)* Colorless gum; [α]^20^_D_ −48.8 (c = 1.11, MeOH); UV (MeOH) *λ*_max_ (log ε) = 214 (4.63), 292 (3.48) nm; IR *ν*_max_ = 3455, 1687, 1625, 1482, 1396 cm^–1^; CD (MeOH) *λ*_max_ (Δε) 222 (−16.4), 256 (+5.09), 288 (−4.0) nm; For ^1^H NMR (400 MHz) and ^13^C NMR (100 MHz) data see Table 2; HRESIMS [M + Na]^+^ *m/z* 583.2310 (calcd for C_34_H_32_O_4_Na, 583.2321).

*3-benzylidene-3,4-dihydro-4-methyl-lH-l,4-benzodiazepine-2,5-dione (**4**)*^1^ H NMR: δ_H_ 7.89, dd, 1 H (7.9, 1.5); 7.54, ddd, 1H (8.1, 7.4, 1.6); 7.34–7.44, overlapped, 5 H; 7.28, ddd, 1 H (7.9, 7.4, 1.1); 7.13, dd, 1 H (8.1, 0.7); 3.11, s, 3 H. ^13^C NMR: δ_C_ 172.4, 169.0, 137.9, 135.3, 134.1, 133.6, 131.8, 130.9, 130.2, 130.2, 126.8, 125.9, 122.0, 36.1.

*Hydrolysis and modified Marfey’s Analysis of **3*** Compound **3** (0.8 mg) was dissolved in 1.0 mL of 6 N HCl and heated to 110 °C for 1 h. The solution was evaporated to remove the trace HCl under vacuum. The divided hydrolysate (0.4 mg each) was treated with 100 *μ*L of 1 N NaHCO_3_ followed by 50 *μ*L of 1% L- or D-FDAA in acetone. The mixture was stirred at 80 °C for 15 min. After quenching by the addition of 50 *μ*L of 2 N HCl, the mixture was analyzed by HPLC to assign the chirality of the amino acid (Linear gradient: 10−40% acetonitrile in 60 minutes). Separately, the standard amino acids L-Phe and D-Phe were derivatized with FDAA in the same manner as **3**.

### Protein expression and purification

The gene encoding 11*β*-HSD1 (Genebank: NC_018912.2) was cloned into the pET-28a vector (Novagen). After the recombinant plasmids were verified by sequencing, the plasmid was transformed into *E. coli* BL21(DE3) (Invitrogen) which were grown in LB medium at 37 ^o^C to an OD600 (0.8–1.0) and induced by 0.4 mM isopropyl -D-thiogalactopyranoside (IPTG) at 20 ^o^C for 16 hours. Bacterial cells were lysed by ultrasonification on ice in a buffer containing 20 mM Tris, pH 8.5, 200 mM NaCl, 5 mM mercaptoethanol, 0.1% TritonX-100, and 5% glycerol. Soluble C-terminally hexa-histidine tagged 11*β*-HSD1 was bound to Ni-agarose affinity resin (Qiagen), washed with a buffer containing 20 mM Tris, pH 8.5, 200 mM NaCl, and 10 mM imidazole, and eluted with a buffer containing 20 mM Tris, pH 8.8, 250 mM NaCl, and 150 mM imidazole. Thrombin (Roche) was added at one unit per 4 mg protein to the elute, which was then dialyzed against 20 mM Tris pH 8.5 and 100 mM NaCl at room temperature for overnight digestion. After thrombin digestion to remove the hexahistidine tag, contaminant proteins were removed by loading the elute onto a second cobalt affinity column equilibrated in the dialyzing buffer and collecting the flow-through. The protein was further purified with anion exchange chromatography, using a linear gradient of 10 mM to 1 M NaCl concentration and size exclusion chromatography at 20 mM Tris pH 8.5 and 200 mM NaCl^22^,^23^.

### Enzyme inhibition assay

The inhibitory effects of 1–8 on 11*β*-HSD1 were measured using recombinant human 11*β*-HSD1 protein through HPLC-MS/MS method^24^,^25^. Briefly, 2.5 *μ*L recombinant 11*β*-HSD1 protein (0.512 mg protein/ml) was added to 100 *μ*L buffer (100 mM KCl, 20 mM NaCl, 20 mM Hepes, pH 7.5) containing NADPH (1 mM) and cortisone (50 μM) with or without various concentrations of compounds. The reactants were incubated at 37 ^o^C for 30 min. After extraction with dichloromethane, evaporation and resolvation with 200 *μ*L methanol, cortisone and cortisol were measured by HPLC-MS/MS and multi-reactions monitoring (MRM) technology. Detection was performed using a Shimadzu LC-20AD HPLC system equipped with an SHIMADZU VP-ODS 150 L × 4.6 mm column connected to a API 4000 LC/MS/MS system. Samples were analyzed after injection and equilibrated with 45% solution A (0.1% formic acid in water) and 55% solution B (Methanol) under isocratic elution with the flow rate of 0.8 mL/min. The HPLC eluent were tested by HPLC-MS/MS using collision energy 33 eV (cortisol) and 30 eV (collision) in positive ion mode. Analyst quantitation optimization wizard was used to optimize the collision energies and other electric lens settings for MRM detections. The inhibition rates were calculated using the concentration ratios of cortisone and cortisol in tubes with or without various compounds.

### Binding affinity assay using microscale thermophoresis

The ability of the purified 11*β*-HSD1 to bind with potential ligands was analyzed using MST. The protein was labeled with the Monolith NT™ Protein Labeling Kit RED (Cat#L001) according to the supplied labeling protocol^26^,^27^. Labelled 11*β*-HSD1 was kept constant at 50 nM, while all samples tested were diluted in a 20 mM HEPES (pH 7.5) and 0.05 (v/v)% Tween-20. Compounds were diluted in 12 dilution steps covering the range of appropriate concentrations. After 10 min incubation at room temperature, samples were loaded into Monolith^TM^ standard-treated capillaries and the thermophoresis was measured at 22 °C after 30 min incubation on a Monolith NT.115 instrument (NanoTemper Technologies, München, Germany). Laser power was set to 40% using 30 seconds on-time and the LED power was set to 100%. The dissociation constrant Kd values were fitted by using the NTAnalysis software (NanoTemper Technologies, München, Germany).

### Differentiation Induction of 3T3-L1

3T3-L1 preadipocytes were purchased from Shanghai Institute of Cell Biology, Chinese Academy of Sciences. Both of them were cultured in DMEM medium (Gibco, USA) containing 10% FBS, penicillin (100 U/mL), streptomycin (100 g/mL) in 5% CO2 at 37 °C. 3T3-L1 preadipocytes were induced to differentiate as previously described. In short, differentiation was induced by adding 0.5 mmol/L 3-isobutyl-1-methyxanthine (Sigma), 10 *μ*g/mL insulin (Sigma), and 1 mmol/L dexamethasone (Sigma) to the medium. After 48 h incubation (d2), the medium was replaced with DMEM containing 10 *μ*g/mL insulin and 50 *μ*M compound 3. On d4, the medium was replaced with DMEM containing compound 3, and then changed back to the same medium every on d6.

### Oil red O staining

Preadipocytes 3T3-L1 were induced to differentiate with or without compounds 3 for 6 days, and then washed twice with PBS, fixed in 3.7% formaldehyde for 1 h and stained with Oil Red O for another 1 h at room temperature. Cells were then washed several times with PBS, and photographed by a light microscope (Olympus, Japan).

### Western Blot Analysis

Cells were treated and then lysed in a radio immune-precipitation assay buffer. Protein concentrations were determined using a BCA protein assay kit (Byontime, Beijing, China) and equalized before loading. Samples were denatured and subjected to electrophoresis in 10% SDS-PAGE gels followed by transfer to PVDF membrane and probed with 11*β*-HSD1 (sc-20175) antibody and *β*-Actin. Blots bands were visualized using the horseradish peroxidase conjugated secondary antibodies and chemiluminescent substrate.

### Molecular Docking

**C**rystal structures of huaman 11*β*-HSD1 (PDB code: 2BEL) was obtained from the Protein Data Bank (http://www.rcsb.org)^28^. The docking was performed by using ICM 3.8.2 modeling software on an Intel i7 4960 processor (MolSoft LLC, San Diego, CA)^29^. Ligand binding pocket residues were selected by using graphical tools in the ICM software, to create the boundaries of the docking search. In the docking calculation, potential energy maps of the receptor were calculated using default parameters. Compounds were imported into ICM and an index file was created. Conformational sampling was based on the Monte Carlo procedure^30^, and finally the lowest-energy and the most favorable orientation of the ligand were selected.

## Additional Information

**How to cite this article**: Sun, W. *et al*. Novel small molecule 11*β*-HSD1 inhibitor from the endophytic fungus *Penicillium commune. Sci. Rep.* **6**, 26418; doi: 10.1038/srep26418 (2016).

## Supplementary Material

Supplementary Information

## Figures and Tables

**Table 1:** NMR data for penicophenones A (**1**) and B (**2**)^a^.

***no***.	**1**		**2**	
	***δ***_**H**_	***δ***_**C**_	***δ***_**H**_	***δ***_**C**_
1		157.3		111.2
2		118.2		160.9
3	7.28 s	130.9		113.8
4		114.1		160.6
5		161.9		116.3
6		110.1	7.58 s	131.1
7	*α* 2.69 dd (16.8, 6.4) *β* 2.20 dd (16.8, 2.8)	24.5	3.75 s	21.54
8	1.88 m	34.5		117.4
9		102.9		114.9
10	*α* 1.09 dd (12.6, 11.3) *β* 2.13 ddd (12.6, 4.8, 1.7)	40.8	6.46 s	100.8
11	4.00 m	65.3		148.1
12	*α* 0.99 dt (12.1, 11.6) *β* 1.81 m	43.1		141.8
13	3.61 dqd (12.5, 6.3, 2.1)	67.7	6.51 s	114.9
14	1.94 s	15.4		203.2
15		204.8	2.50 s	26.3
16	2.33 s	26.3	2.10 s	16.1
17	0.73 d (7.1)	15.4	3.52 s	56.74
18	0.87 d (6.3)	21.7	3.66 s	55.5

^a^400 MHz for ^1^H NMR and 100 MHz for ^13^C NMR (**1** in CD_3_OD and **2** in “DMSO-*d*6”).

**Table 2:** NMR data for Penicopeptide A (**3**)^a^.

***no***.	***δ***_**H**_	***δ***_**C**_	***no***.	***δ***_**H**_	***δ***_**C**_
1		172.3	18		171.1
2	4.33 dd (10.6, 7.0)	69.7	19	4.43 t (7.6)	57.9
3	2.79 dd (13.5, 6.9) 2.66 dd (13.5, 10.8)	35.2	20	3.40 dd (14.5, 7.9) 3.25 dd (14.5, 7.3)	32.9
4		137.1	21		138.1
5	7.02 d (7.0)	130.0	22	7.22 m	130.0
6	7.22 m	129.8	23	7.22 m	129.6
7	7.22 m	128.3	24	7.17 m	127.8
8	7.22 m	129.8	25	7.22 m	129.6
9	7.02 d	130.0	26	7.22 m	130.0
10	2.90 s	39.8	27	3.07 s	29.6
11		168.2	28		170.7
12		127.8	29		128.3
13	7.96 brd (7.9)	132.3	30	7.82 brd (7.8)	131.8
14	7.34 t (7.5)	125.9	31	7.28 m	126.0
15	7.59 brd (7.8)	134.2	32	7.51 brt (7.1)	133.8
16	7.17 d (8.2)	121.6	33	7.07 d (8.2)	122.0
17		137.0	34		138.0

^a^400 MHz for ^1^H NMR and 100 MHz for ^13^C NMR in CD_3_OD.

**Table 3:** Binding affinity and inhibitory activities of compounds **1**–**8**.

**Compound**	**Inhibitory activities against 11*****β*****-HSD1**	**Dissociation constant with 11*****β*****-HSD1**
	**Kd a (*μ*M)**	**IC50 (*μ*M)**
**1**	n.b.b	n.i. c
**2**	n.b.	n.i.
**3**	82.1 ± 5.01	9.07 ± 0.61
**4**	n.b.	n.i.
**5**	420.51 ± 12.11	45.51 ± 7.82
**6**	513.14 ± 10.61	68.05 ± 7. 84
**7**	n.b.	n.i.
**8**	n.b.	n.i.

a The Kd value is automatic calculated by the curve fitting, and presents as means ± SD for three experiments.

b n.b. is no clear binding detected in the MST measurement.

c n.i. is no inhibition detected in the experiments (IC_50_ > 200 *μ*M).
