# Supplementary material for: Novel small molecule 11β-HSD1 inhibitor from the endophytic fungus Penicillium commune
Source: Sci Rep. 2016 May 19;6:26418. doi: 10.1038/srep26418 (PMC4872216; doi:10.1038/srep26418)
Supplement: Supplementary Information [file srep26418-s1.pdf]

Novel small molecule 11β-HSD1 inhibitor from the endophytic fungus Penicillium commune

Weiguang Sun [1], Xintao Chen [1], Qingyi Tong [1], Hucheng Zhu [1], Yan He[1], Liang Lei[2], Yongbo Xue [1], Guangmin Yao[1], Zengwei Luo[1], Jianping Wang[1,]*, Hua Li[1,]* & Yonghui Zhang[1,]*

[1] Hubei Key Laboratory of Natural Medicinal Chemistry and Resource Evaluation, School of Pharmacy, Tongji Medical College, Huazhong University of Science and Technology, Wuhan 430030, China

[2] Puai Hospital Affliated to Tongji Medical College, Huazhong University of Science and Technology, Wuhan 430030, People's Republic of China

* **Corresponding Authors**

* Tel./fax: +86-027-83692892

Emails: zhangyh@mails.tjmu.edu.cn (Y.Z.);

li_hua@hust.edu.cn (H. L.)

jpwang1001@163.com (J. P.)

Content

Fungal Material..1
HRESIMS spectrum for 1..3
CD spectrum for 1……………………………………………………………………..2
^{1}H NMR spectrum for 1..4
^{13}C NMR and DEPT spectra for 1..4
HSQC spectrum for 1..5
HMBC spectrum for 1..5
^{1}H-^{1}H COSY spectrum for 1..6
NOESY spectrum for 1..6
UV (MeOH) spectrum for 1..7
IR spectrum for 1..8
HRESIMS for spectrum for 2..8
^{1}H NMR spectrum for 2..9
^{13}C NMR and DEPT spectra for 2..9
HSQC spectrum for 2..10
HMBC spectrum for 2..10
UV (MeOH) spectrum for 2..11
IR spectrum for 2..11
HRESIMS spectrum for 3..12
^{1}H NMR spectrum for 3..12
^{13}C NMR and DEPT spectra for 3..13
HSQC spectrum for 3..13
HMBC spectrum for 3..14
^{1}H-^{1}H COSY spectrum for 3..14
UV (MeOH) spectrum for 3..15
IR spectrum for 3..15
CD spectrum for 3..16
^{1}H NMR spectrum for 4..16
^{13}C NMR and DEPT spectra for 4..17
Binding conformations of compound 3 bound to 11β-HSD1 comparing with carbenoxolone generated by virtual ligand docking..……………………………………………………………..18

Fungal Material. The fungal strain *P.commune* was isolated from the *Vitis vinifera*, collected at Qingshui County in Gansu province of China, in July 2007. The 16S RNA sequence data for this strain was listed as follows:

AAGAGGCTCTGGGTCCACCTCCCACCCGTGTTTATTTTACCTTGTTGCTTCGGCGGGCCCGCCTTAACTGGCCGC
CGGGGGGCTTACGCCCCCGGGCCCGCGCCCGCCGAAGACACCCTCGAACTCTGTCTGAAGATTGAAGTCTGAGTG
AAAATATAAATTATTTAAAACTTTCAACAACGGATCTCTTGGTTCCGGCATCGATGAAGAACGCAGCGAAATGCG
ATACGTAATGTGAATTGCAAATTCAGTGAATCATCGAGTCTTTGAACGCACATTGCGCCCCCTGGTATTCCGGGGG
GCATGCCTGTCCGAGCGTCATTGCTGCCCTCAAGCCCGGCTTGTGTGTTGGGCCCCGTCCCCCGATCTCCGGGGGA
CGGGCCCGAAAGGCAGCGGCGGCACCGCGTCCGGTCCTCGAGCGTATGGGGCTTTGTCACCCGCTCTGTAGGCCC
GGCCGGCGCTTGCCGATCAACCCAAATTTTTATCCAGGTTGACCTCGGATCAGGTAGGGATACCCGCTGAACTTAA
GCATATCAATAAGCGGAGGAGGGGGGGCGGGGGGGCAAAGCTCCCCCCGGGGGGGTGTGTTCTTTTGTGGTTTGG
GGGGGCGGCGGGTCAGTCGGAGCTGGGGGGGGGGGGAGGTTCCGGGGCGGGGCGCCCCGAGAACCCCCAATTAT
TTTGTGAAAAAGAAAGTCGGGGCGAAAAAATAATTTTTTATTCCTCTCACCACGCTCTCTGGCCCCGCTTCTATGA
AAAAGCGCGAAAGGATACTAATGGGATTGGAATTCTTGAATATCTGTCTTTTAAGACATTTGCCCGCCCCTACCGT
GGCGGAGCGACCAGAGCGATGGCAGCTCAGCGCCTTGACGCGGGGCAGTGCGCATCTCCCCGCAATCTCGAGGAA
CGCCAACGATCGGTCTCACGCATCTGCCTTGAACGGTTGTATGTCGCCGCGTTGGCAACCCGAGTTTTCAGTGAAC
TTGTTCAGAGATCCGGAACTAGCATCAAACGGGAA

Conformational analyses of **3** were carried out whereby both BALLOON and confab programs. The BALLOON program explores conformational spaces with genetic algorithm, and synchronously, and the confab program systematically generates diverse low energy conformations that are proposed to be close to crystal structures. The conformations generated by the above programs were assembled together via the removal of duplicated conformations whose root mean square (RMS) distance was less than 0.5 Å. Semi-empirical PM3 quantum mechanical geometry optimizations were fulfilled on conformations through the Gaussian 09 program. Duplicated conformations after geometry optimization were subsequently identified and disposed. Remaining conformations were further optimized at B3LYP/6-31G* level of theory in methanol solvent with IEFPCM3 solvation model using Gaussian 09 program, and duplicated conformations emerging after these calculations were removed according to the same RMS criteria above. Harmonic vibrational frequencies were performed to establish the stability of the finally obtained conformers. Six conformations were finally obtained, including **3a-3f**, and most of them were asymmetrical (**3b**, **3d**, and **3e;** 62.34%). Therefore, we think that the asymmetrical comformation of **3** lead to the different NMR data of two units of the dipeptide Phenylalanine-Anthranilic Acid.

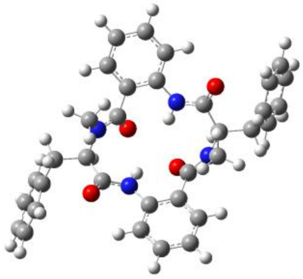	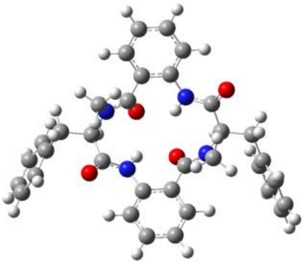	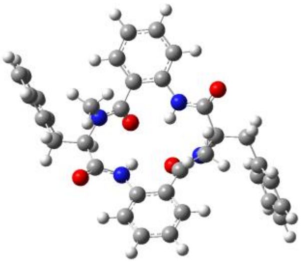
**3a** (16.28%) symmetrical	**3b** (15.26%) asymmetrical	**3c** (10.69%) symmetrical
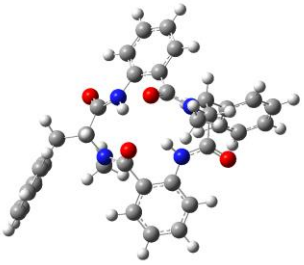	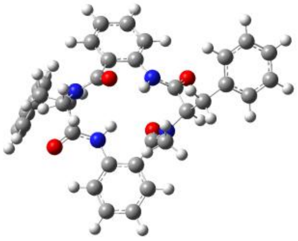	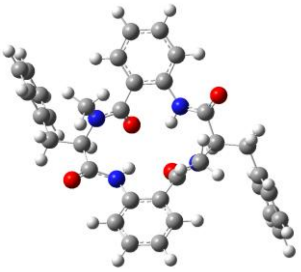
**3d** (6.22%) asymmetrical	**3e** (40.86%) asymmetrical	**3f** (10.69%) symmetrical

HRESIMS spectrum for 1

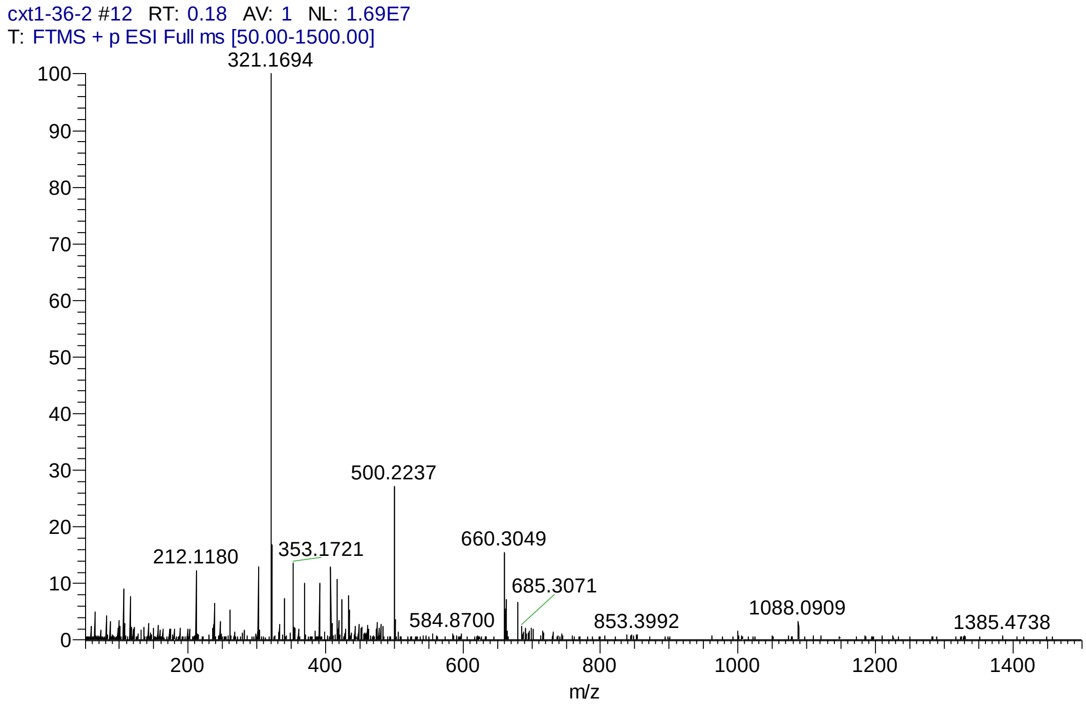

CD spectrum for 1

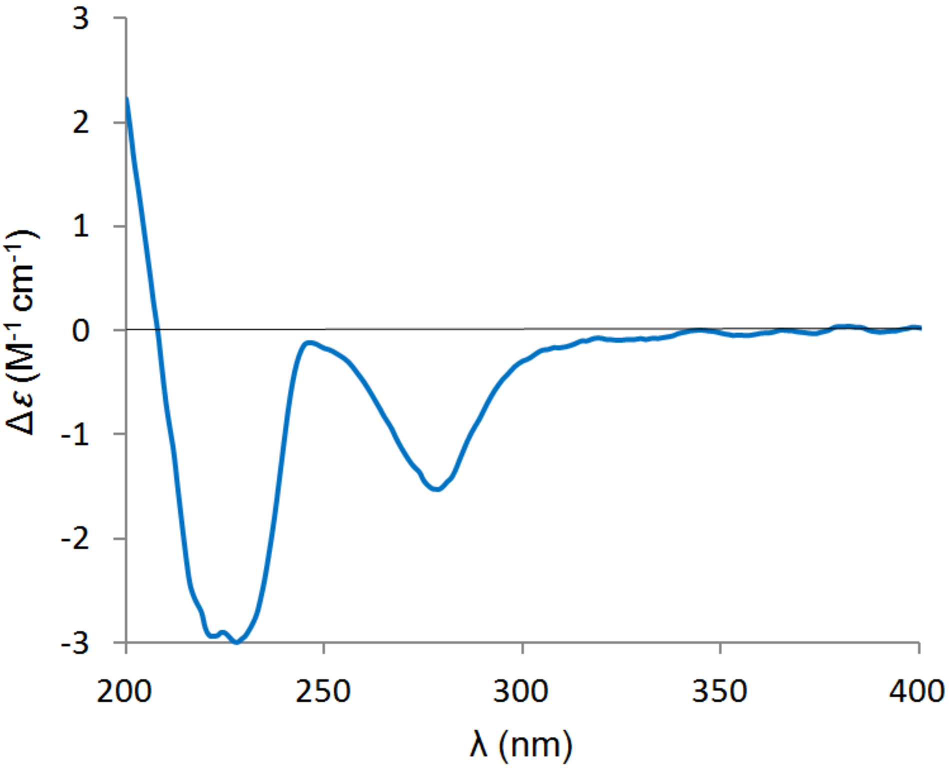

^{1}H NMR spectrum for 1

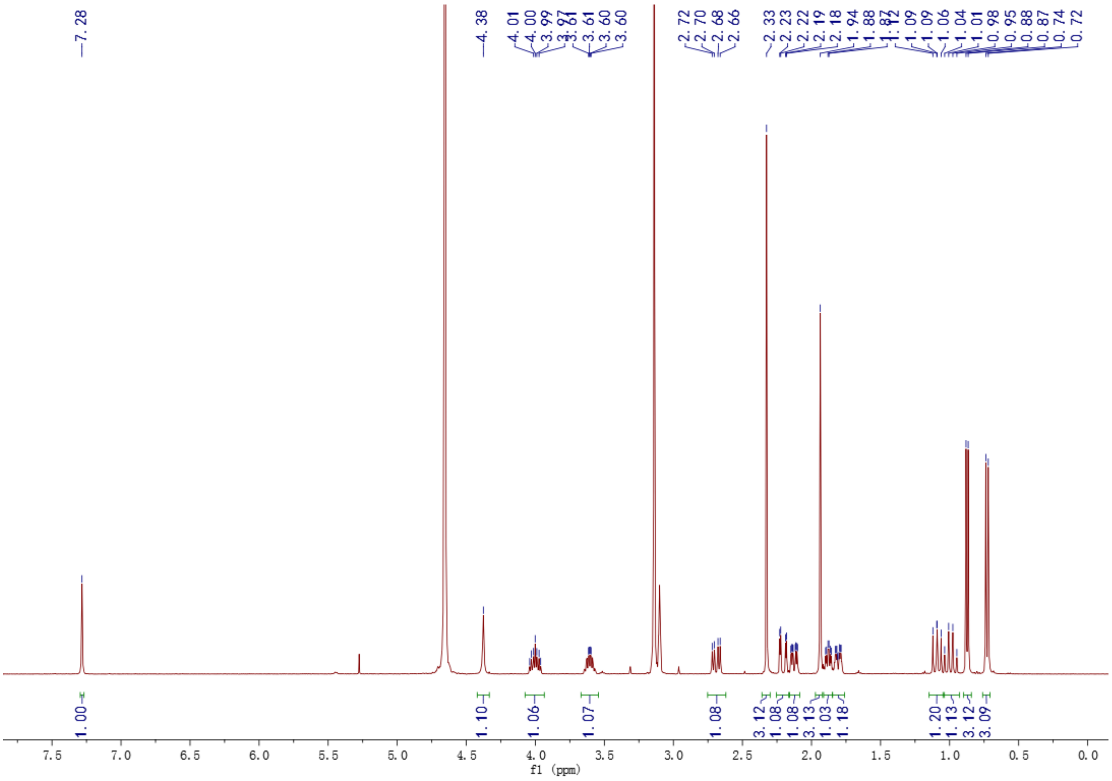

^{13}C NMR and DEPT spectra for 1

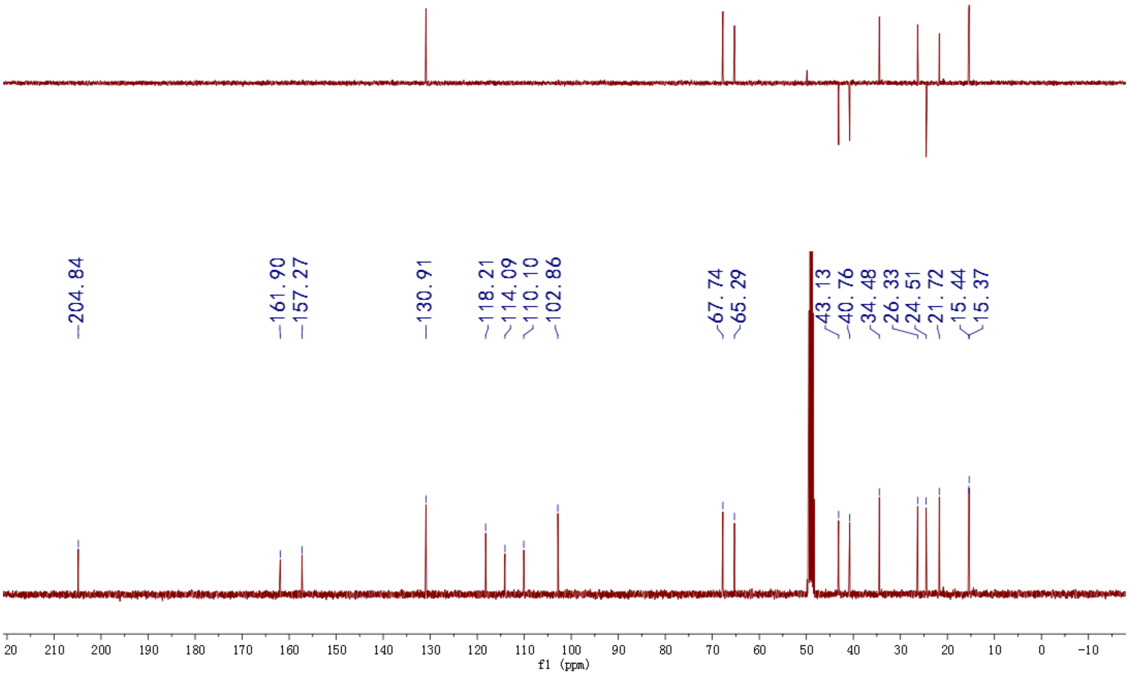

HSQC spectrum for 1

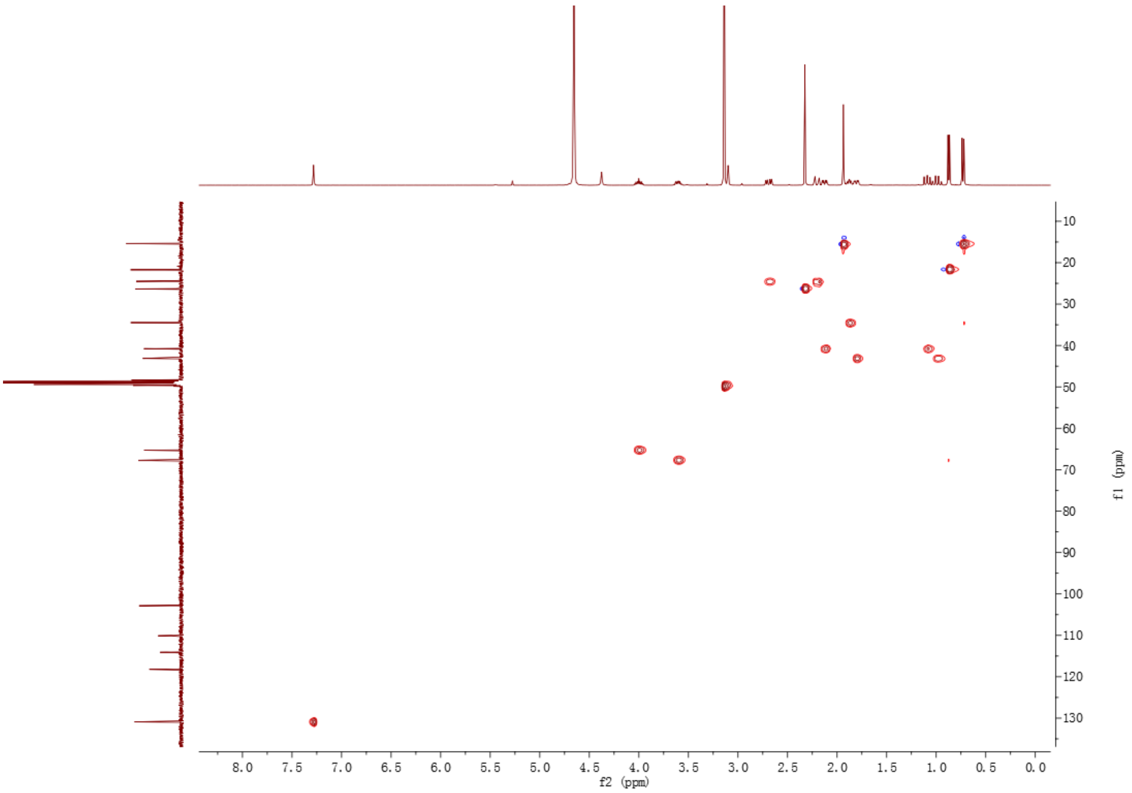

HMBC spectrum for 1

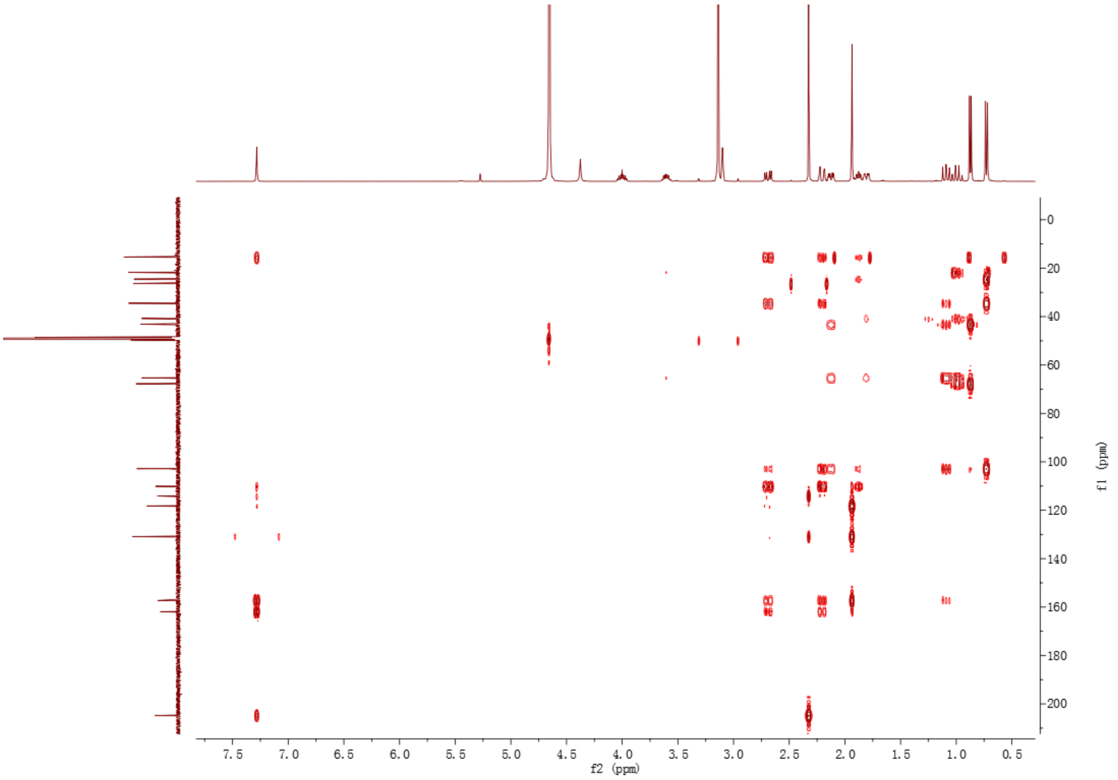

^{1}H-^{1}H COSY spectrum for 1

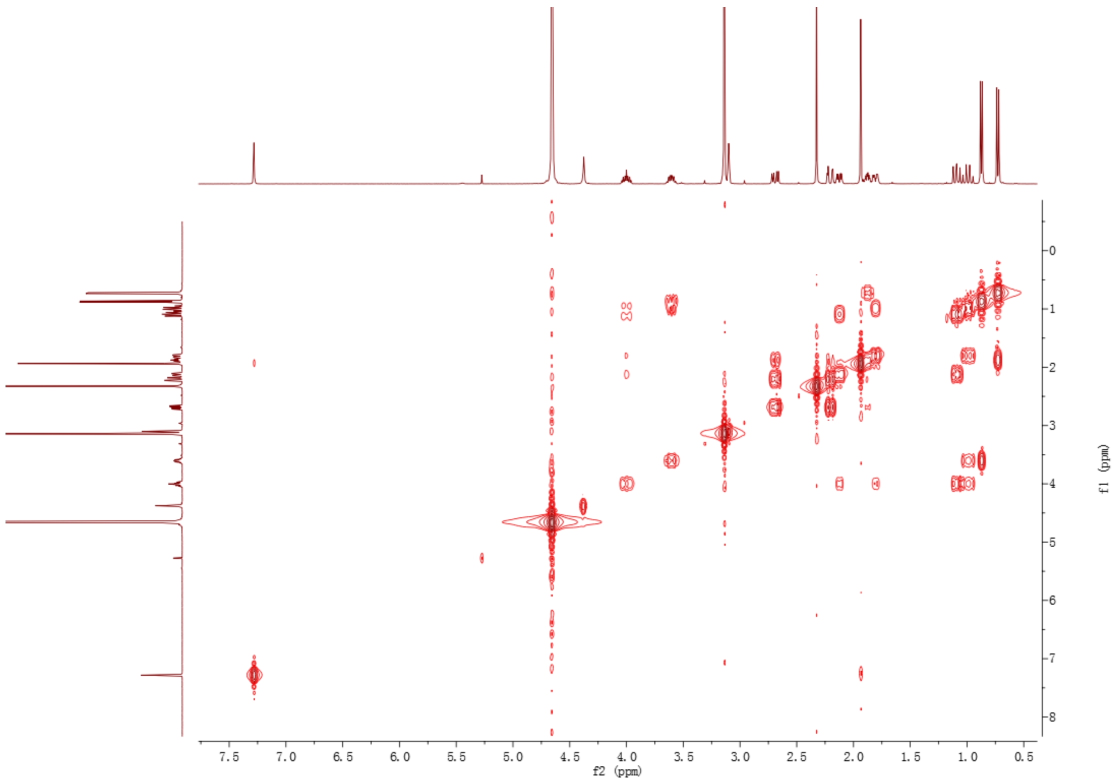

NOESY spectrum for 1

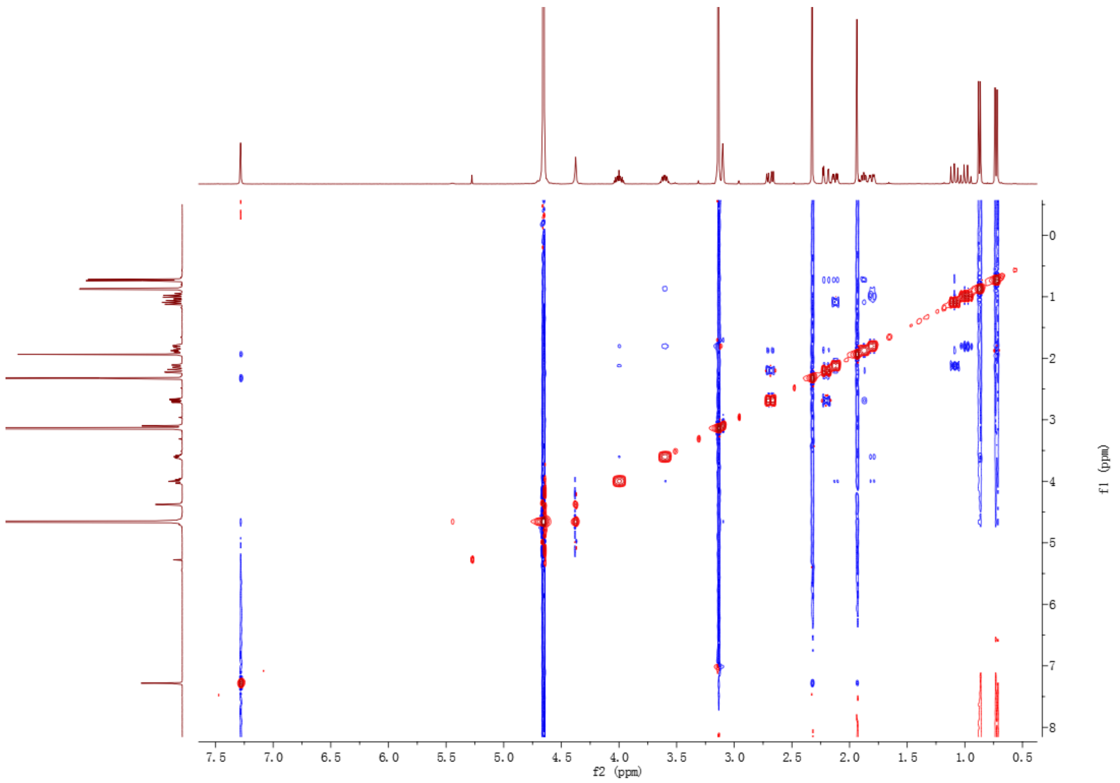

UV (MeOH) spectrum for 1

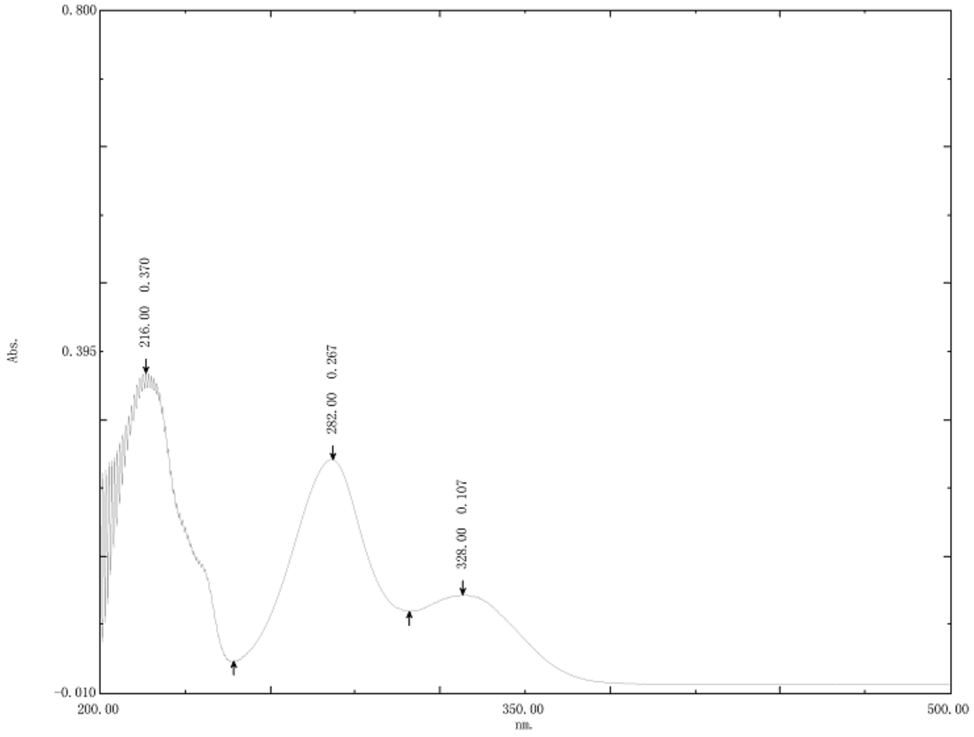

IR spectrum for 1

E:\同济医学院\张勇慧\20141024\cxt36-2.0

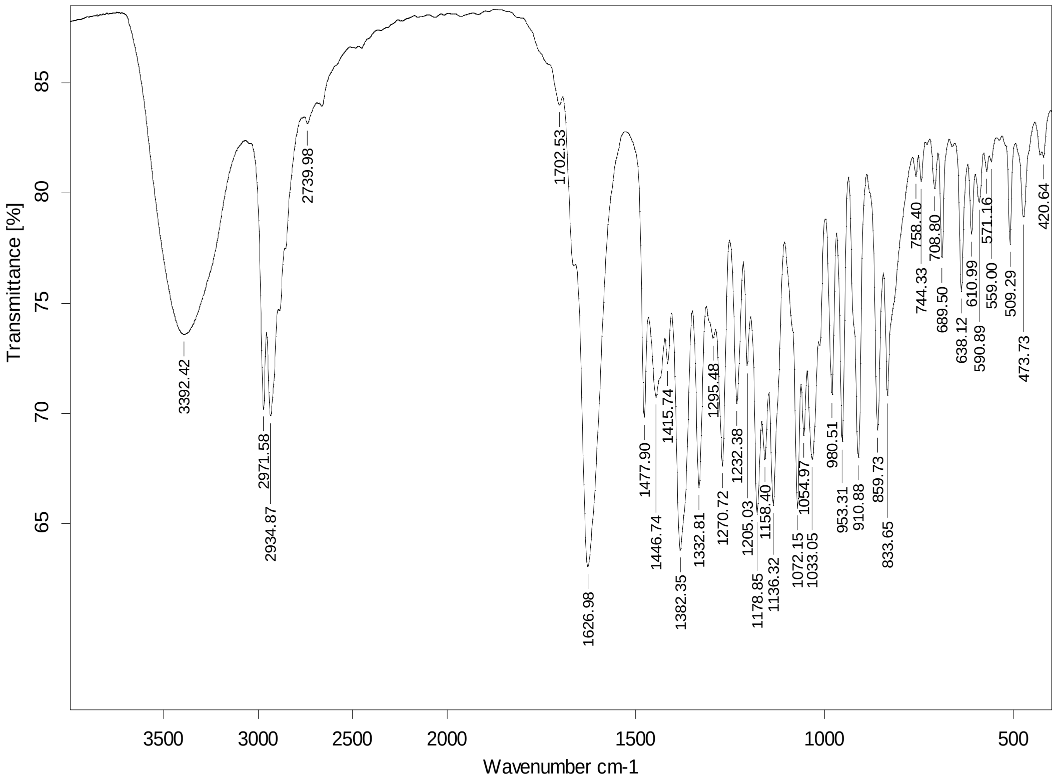

HRESIMS for spectrum for 2

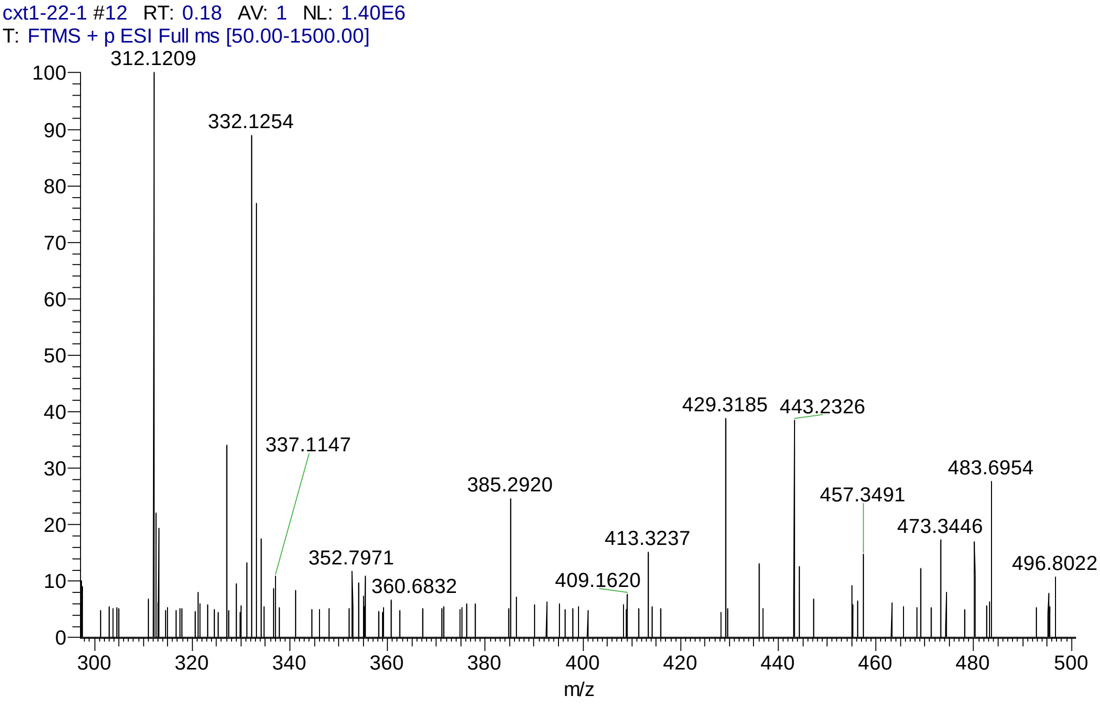

^{1}H NMR spectrum for 2

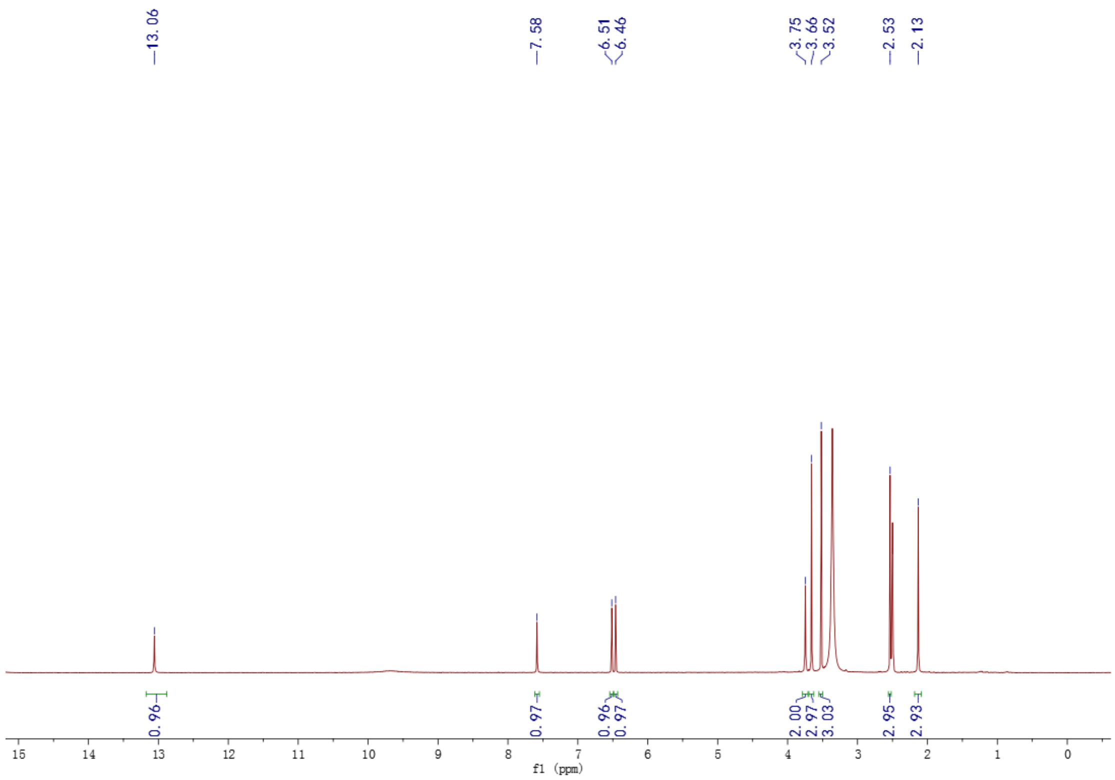

^{13}C NMR and DEPT spectra for 2

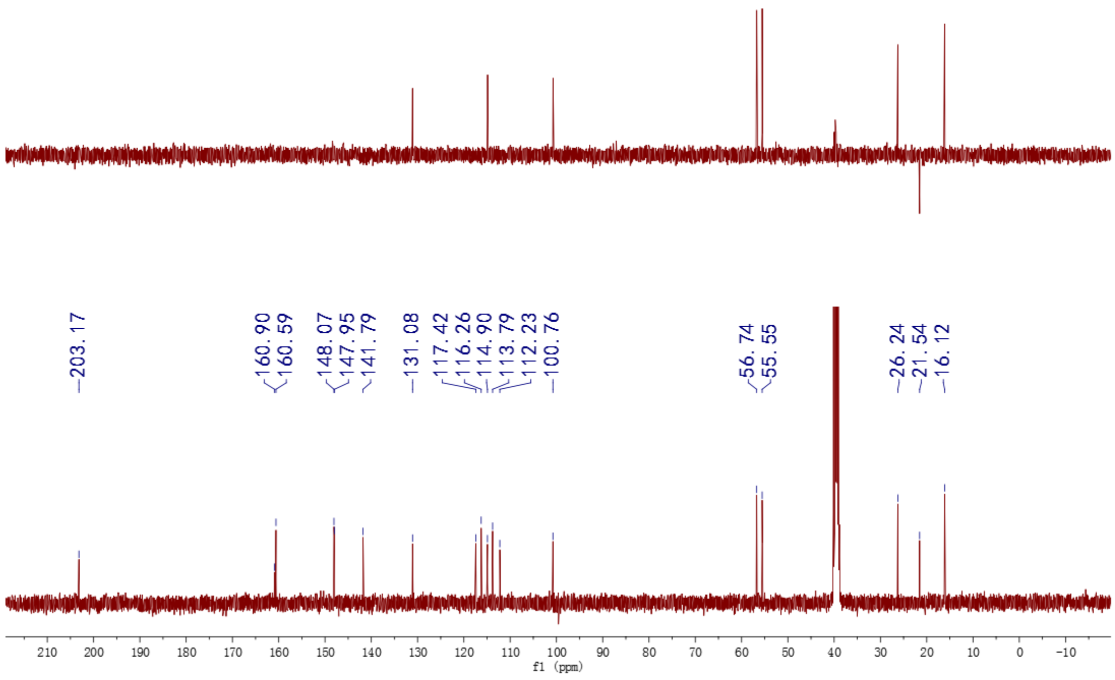

HSQC spectrum for 2

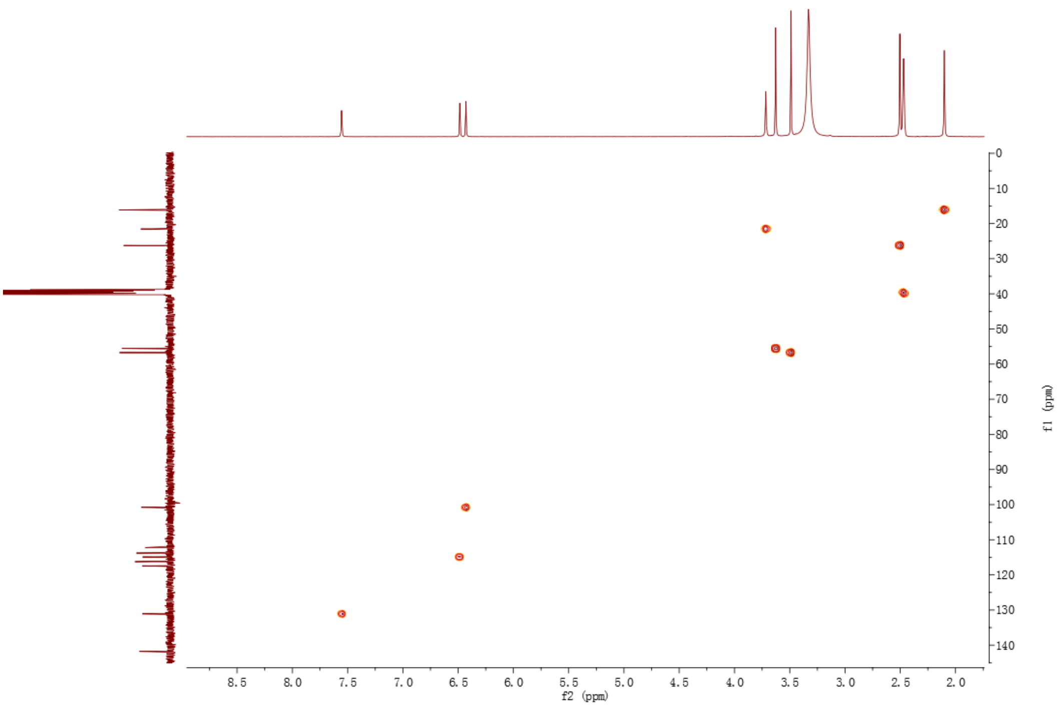

HMBC spectrum for 2

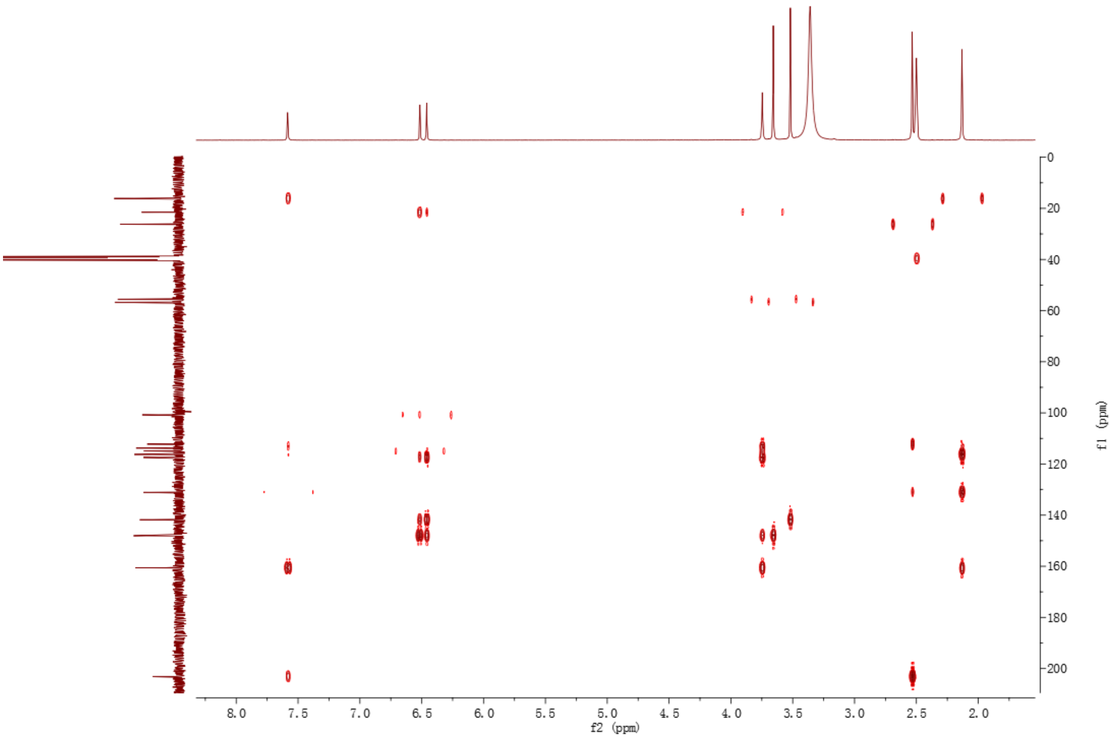

UV (MeOH) spectrum for 2

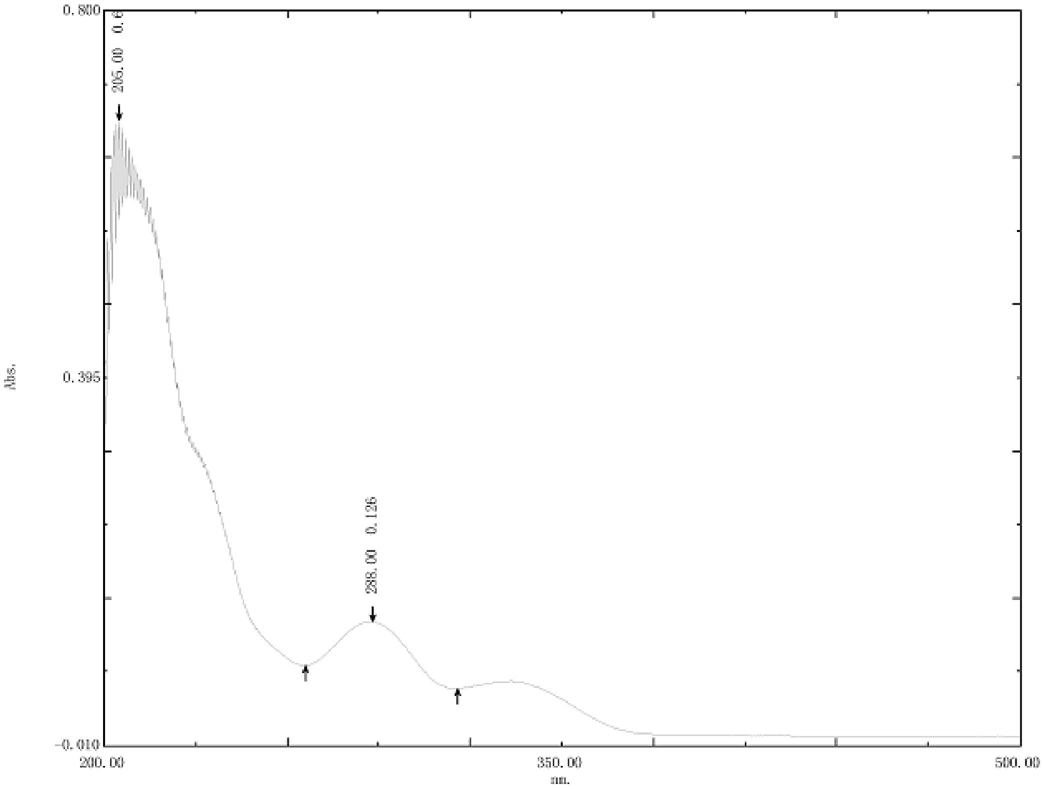

IR spectrum for 2

E:\同济医学院\张勇慧\20141024\cxt22-1.0

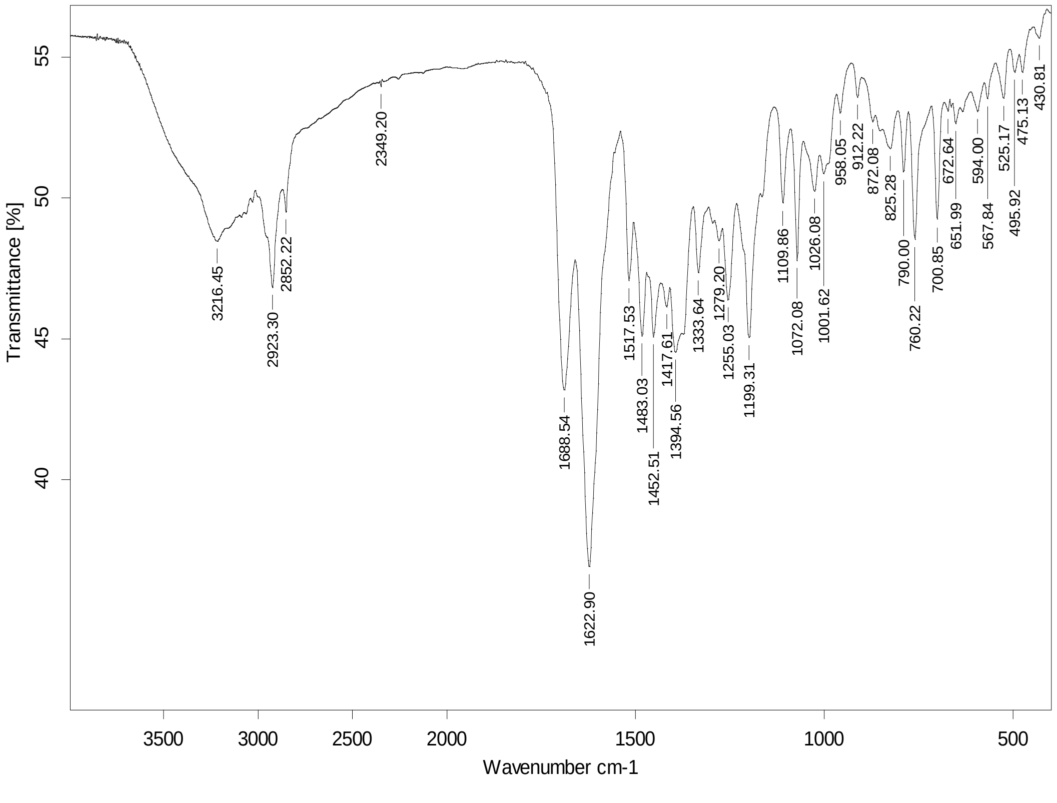

HRESIMS spectrum for 3

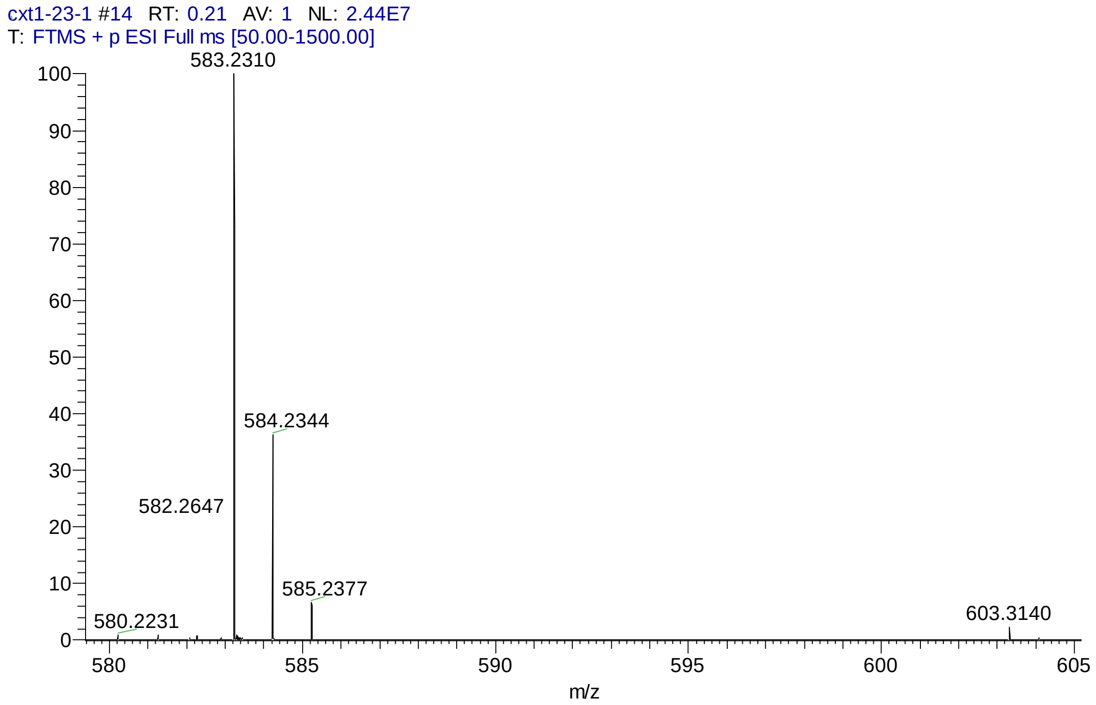

¹H NMR spectrum for 3

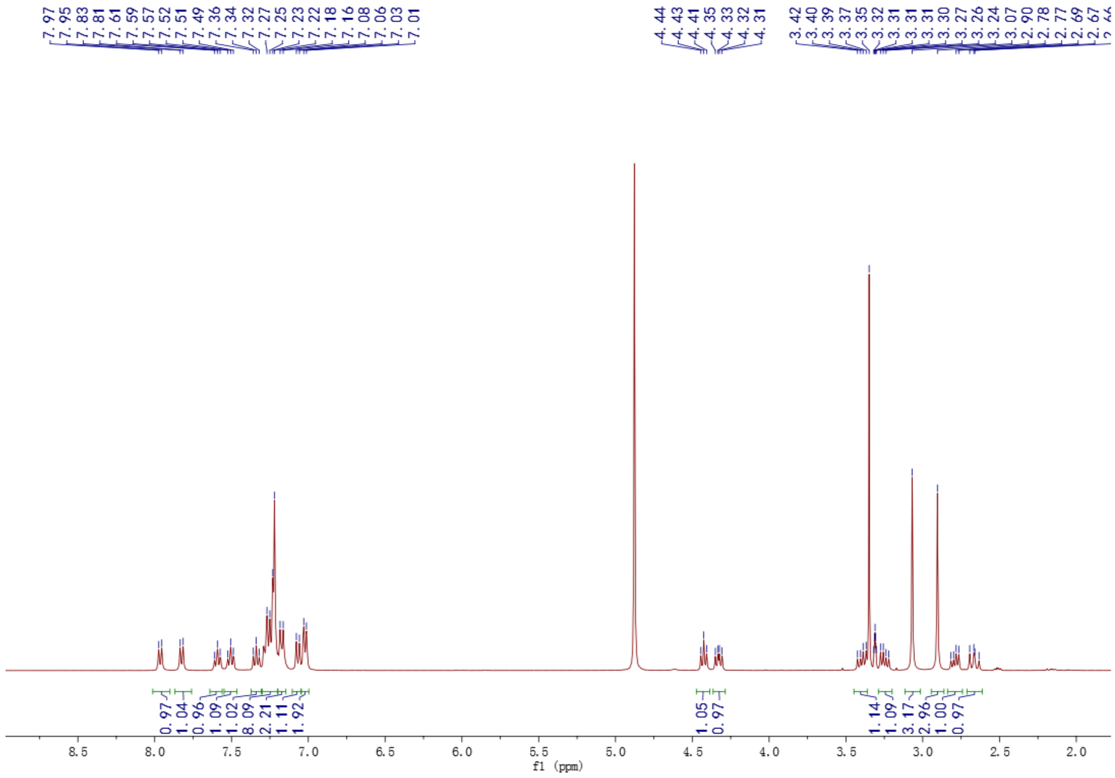

^{13}C NMR and DEPT spectra for 3

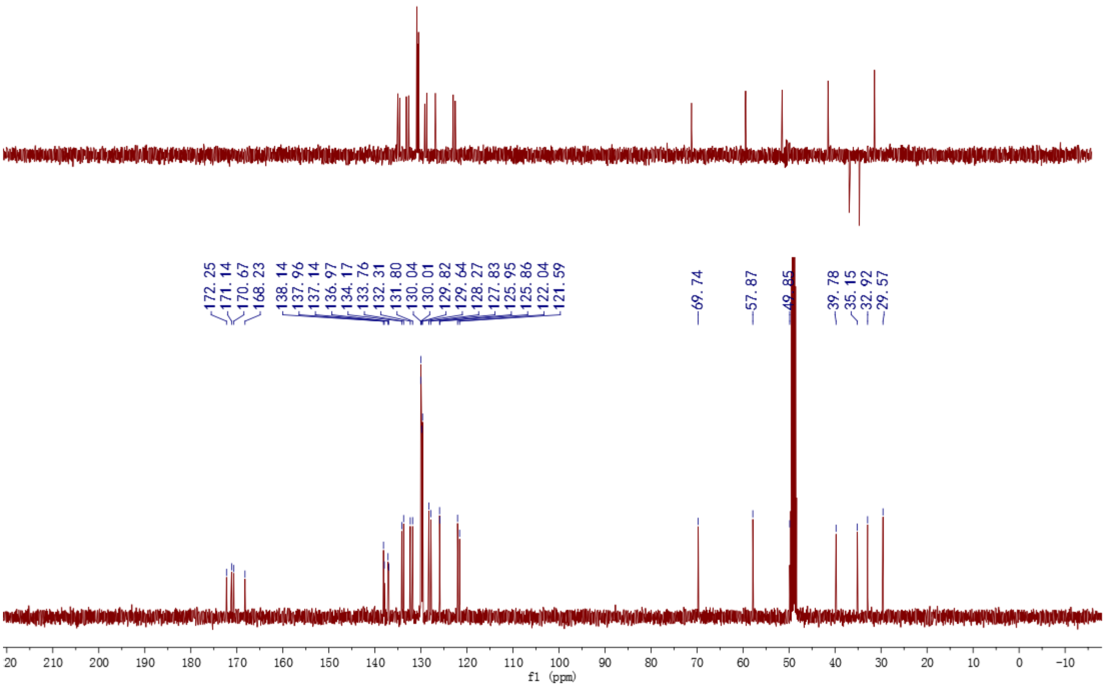

HSQC spectrum for 3

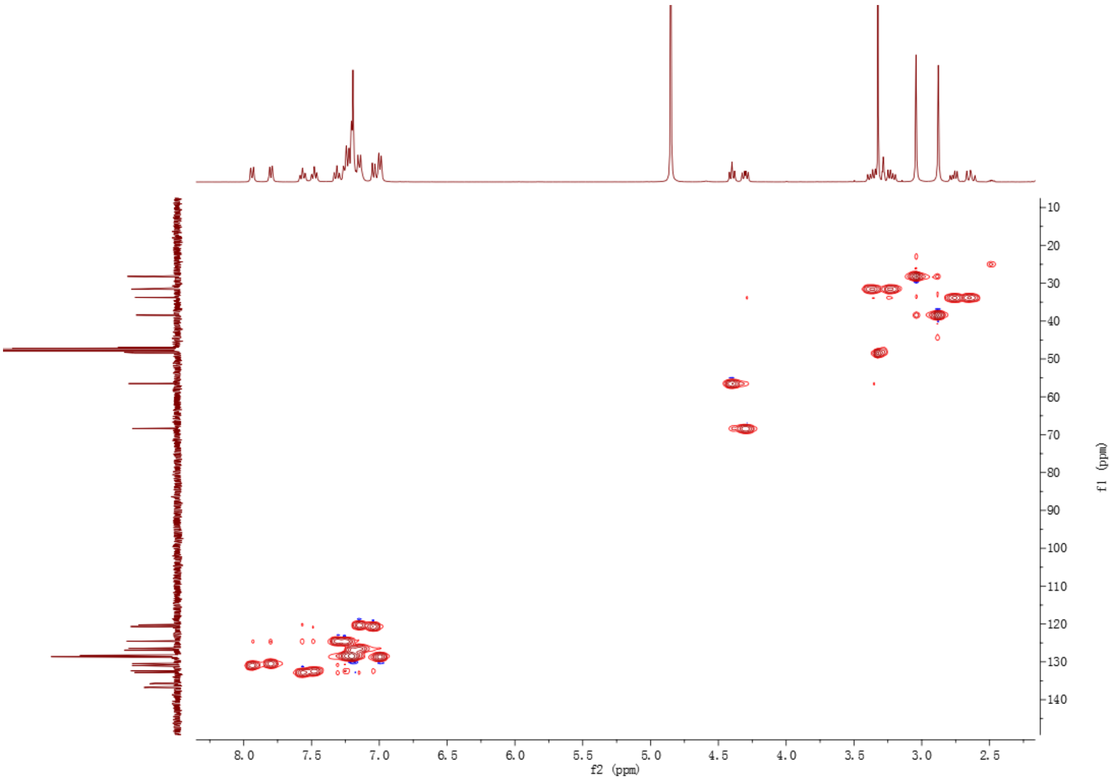

HMBC spectrum for 3

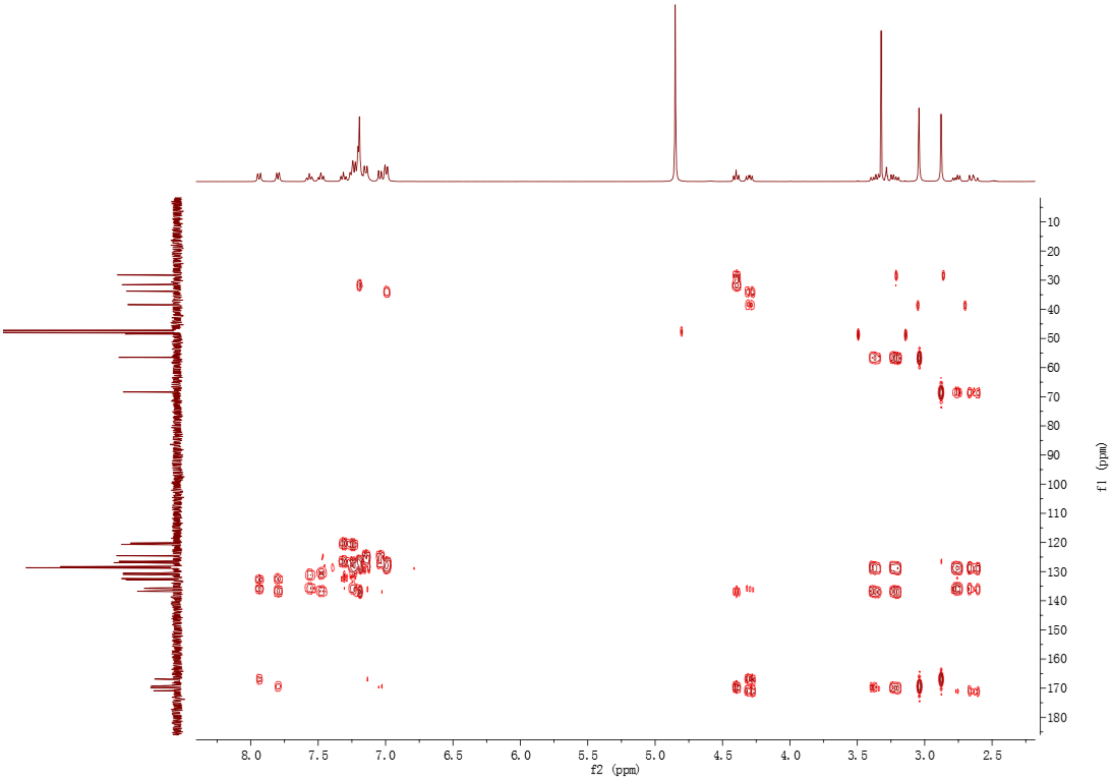

^{1}H-^{1}H COSY spectrum for 3

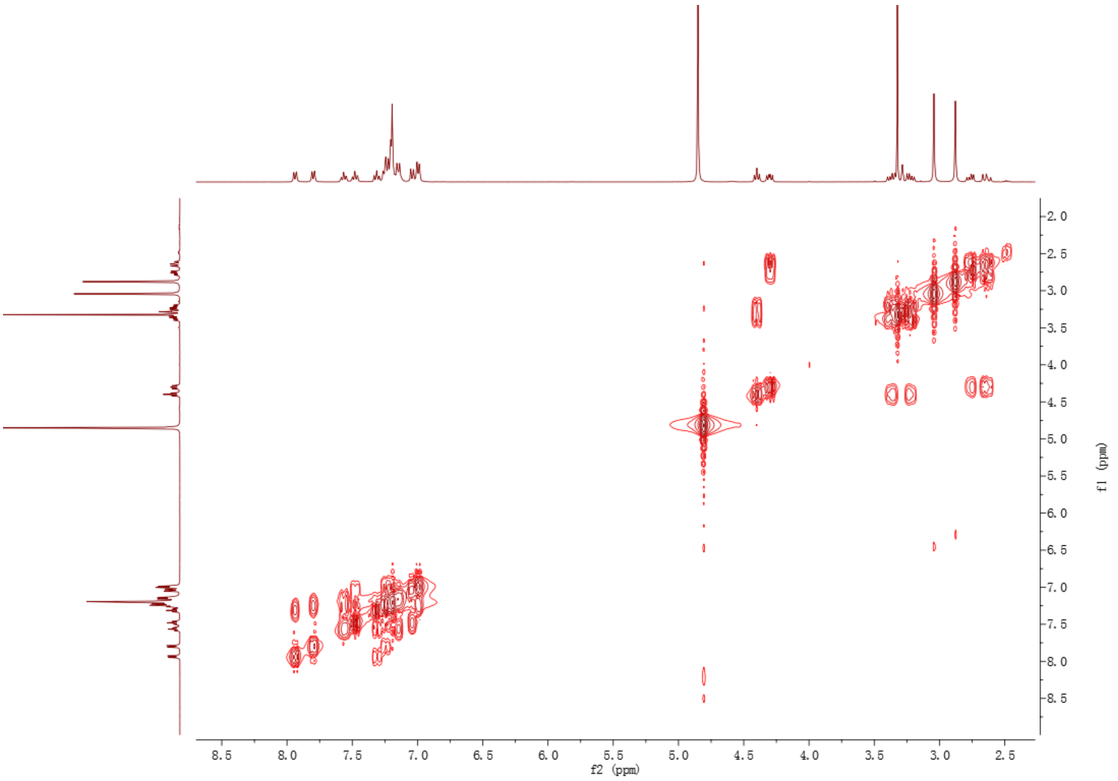

UV (MeOH) spectrum for 3

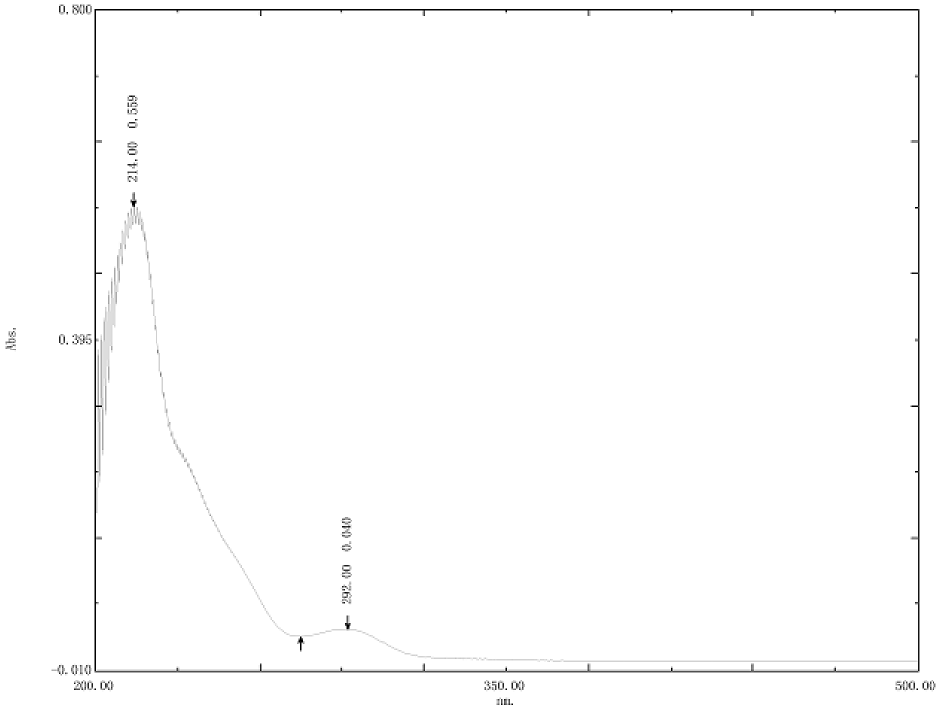

IR spectrum for 3

E:\同济医学院\张勇慧\20141024\cxt23-1.0

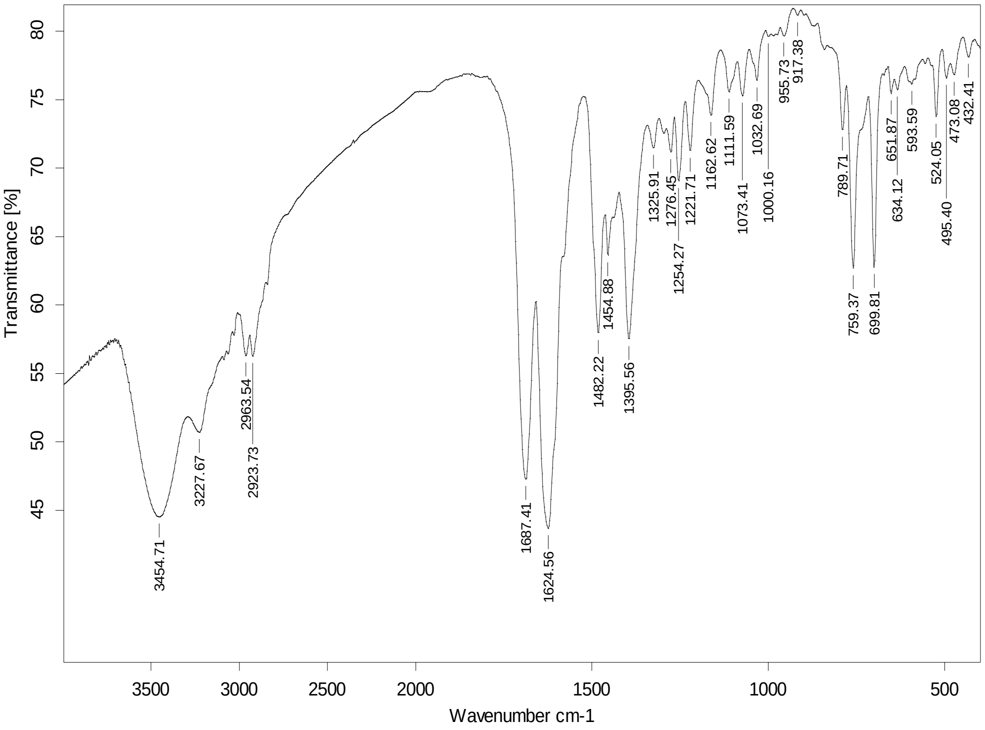

CD spectrum for 3

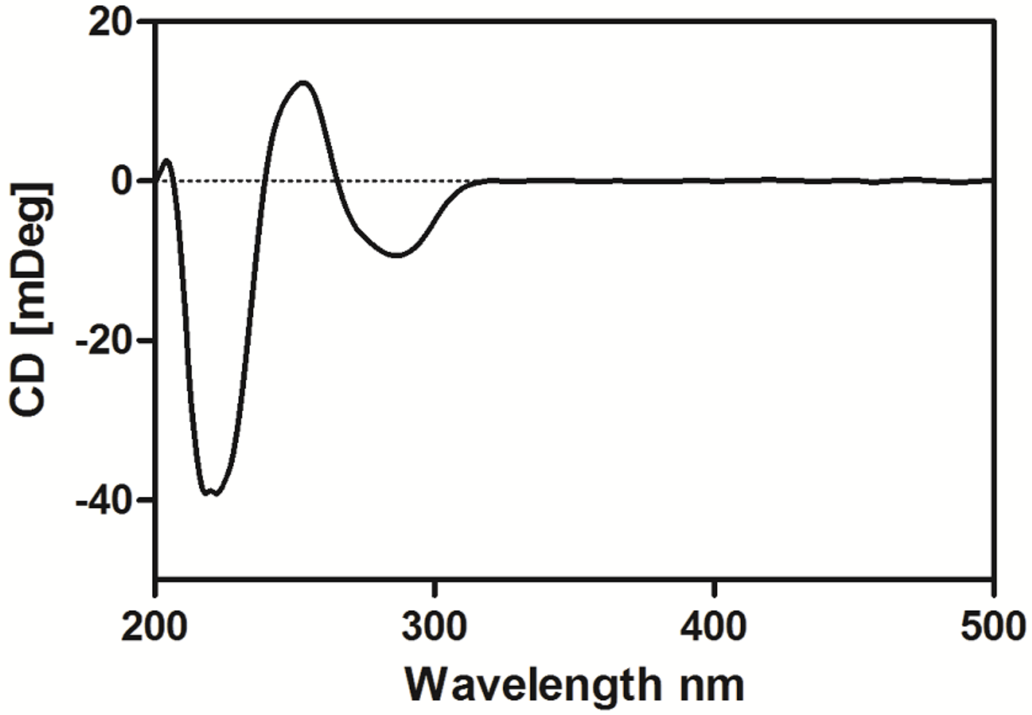

^{1}H NMR spectrum for 4

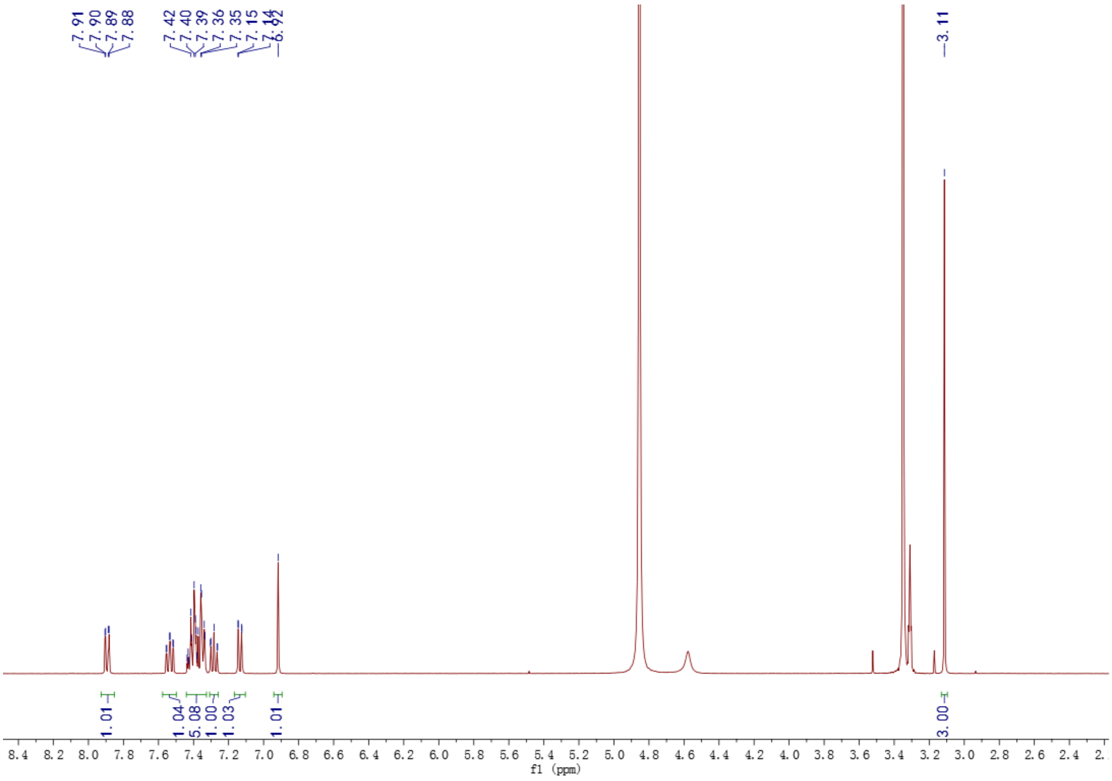

^{13}C NMR and DEPT spectra for 4

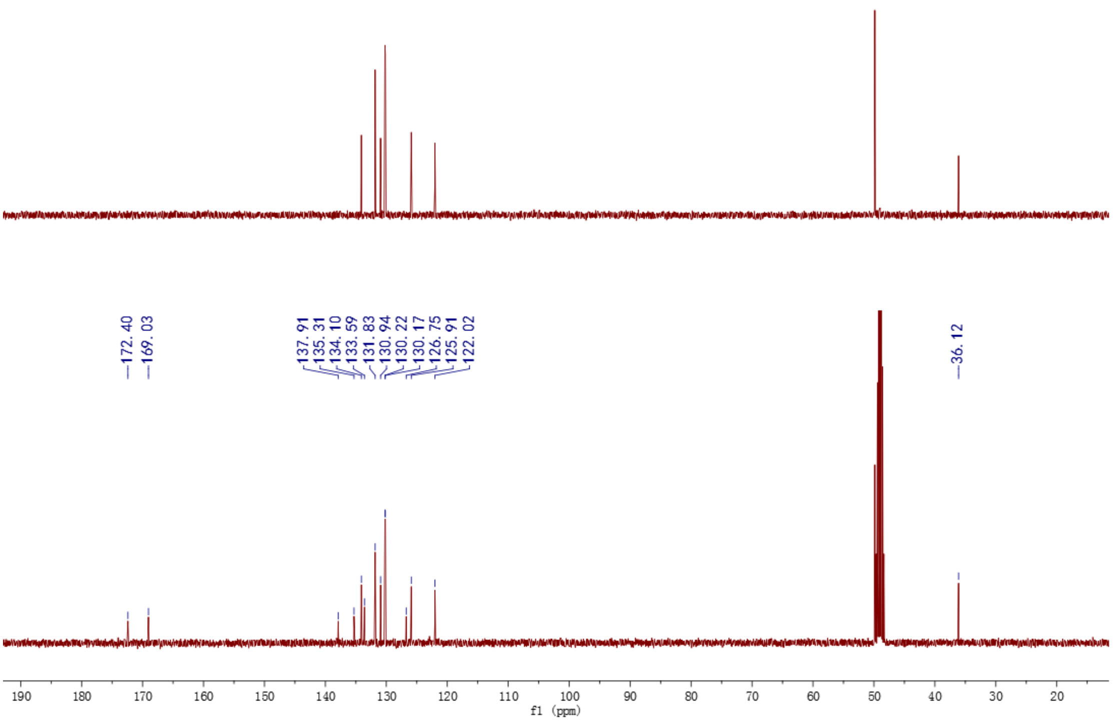

The determination of the absolute configuration of Phe residue in 3

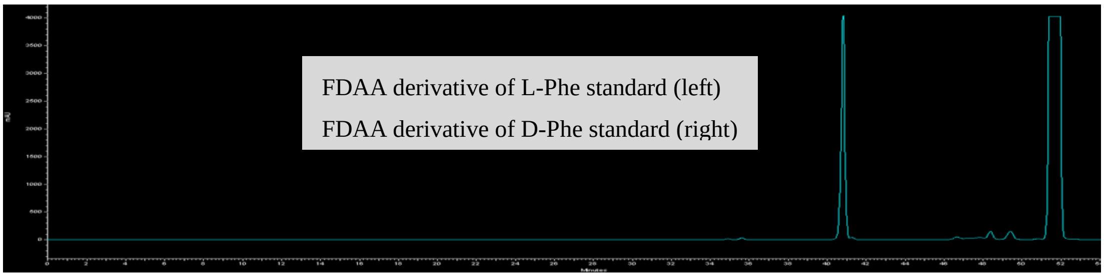

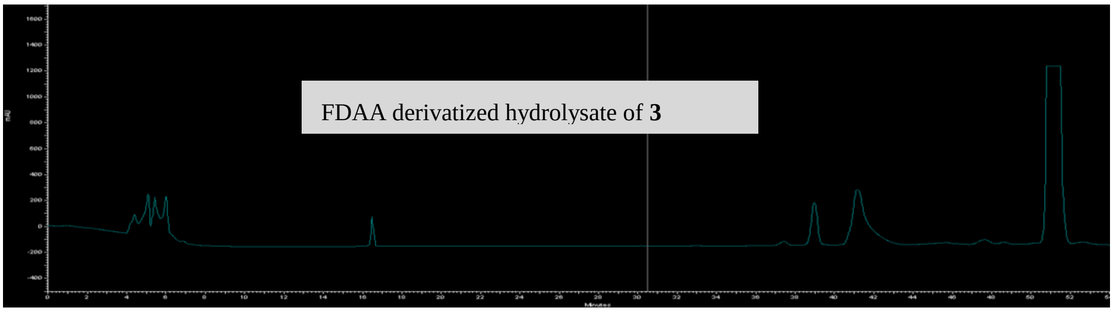

Binding conformations of compound 3 bound to 11β-HSD1 comparing with carbenoxolone generated by virtual ligand docking.

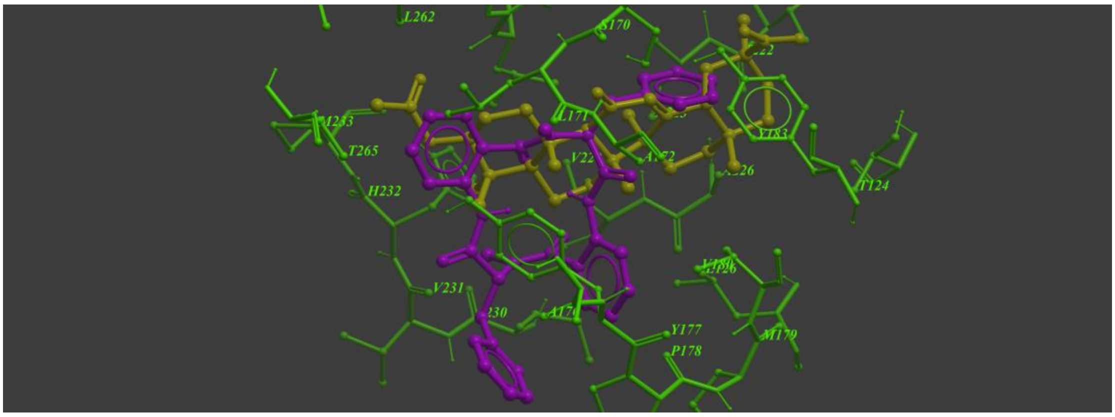

The ball-and-stick model was chosen to show compound **3** (purple), carbenoxolone (yellow) and active site of enzyme (green).
